# Identifying the Hot Spot Residues of the SARS-CoV-2 Main Protease Using MM-PBSA and Multiple Force Fields

**DOI:** 10.3390/life12010054

**Published:** 2021-12-31

**Authors:** Jinyoung Byun, Juyong Lee

**Affiliations:** Department of Chemistry, Division of Chemistry and Biochemistry, Kangwon National University, Chuncheon 24341, Korea; byunjy0614@kangwon.ac.kr

**Keywords:** SARS-CoV-2, main protease, molecular dynamics, multiple force fields, MM-PBSA, ligand binding, absolute binding free energy

## Abstract

In this study, we investigated the binding affinities between the main protease of SARS-CoV-2 virus ($M^{\mathrm{pro}}$) and its various ligands to identify the hot spot residues of the protease. To benchmark the influence of various force fields on hot spot residue identification and binding free energy calculation, we performed MD simulations followed by MM-PBSA analysis with three different force fields: CHARMM36, AMBER99SB, and GROMOS54a7. We performed MD simulations with 100 ns for 11 protein–ligand complexes. From the series of MD simulations and MM-PBSA calculations, it is identified that the MM-PBSA estimations using different force fields are weakly correlated to each other. From a comparison between the force fields, AMBER99SB and GROMOS54a7 results are fairly correlated while CHARMM36 results show weak or almost no correlations with the others. Our results suggest that MM-PBSA analysis results strongly depend on force fields and should be interpreted carefully. Additionally, we identified the hot spot residues of $M^{\mathrm{pro}}$, which play critical roles in ligand binding through energy decomposition analysis. It is identified that the residues of the S4 subsite of the binding site, N142, M165, and R188, contribute strongly to ligand binding. In addition, the terminal residues, D295, R298, and Q299 are identified to have attractive interactions with ligands via electrostatic and solvation energy. We believe that our findings will help facilitate developing the novel inhibitors of SARS-CoV-2.

## 1. Introduction

The new type of coronavirus, the SARS-CoV-2 virus, outbroke in China at the end of 2019. Since its first outbreak, it has led to more than 265 million infections and 5.25 million deaths as of November 2021 [1,2]. Although many vaccines are approved and being developed, no small molecule drug, which has a molecular weight under 500 amu, has been approved by FDA yet as of November 2021 [3,4]. The SARS-CoV-2 genome encodes more than 20 proteins, which include the main protease ($M^{\mathrm{pro}}$) that shares 96.1% similarity with 3CLP of SARS-CoV [5]. $M^{\mathrm{pro}}$ plays an important role in SARS virus replication and transcription. When the mRNA of the virus is translated into a polyprotein chain, $M^{\mathrm{pro}}$ is first autocleaved to become a mature enzyme, which in turn cleaves all of the 11 remaining downstream nonstructural proteins of the polyproteins to polypeptides, which are required for the replication process of the virus [5].

$M^{\mathrm{pro}}$ of SARS-CoV-2 is essential for viral replication and is considered a promising target for drug development [6,7,8]. Based on this assumption, various studies have been performed to find potent inhibitors targeting $M^{\mathrm{pro}}$ [7,9]. Thus, it is critical to evaluate the binding free energy between a ligand and its receptor both accurately and efficiently to accelerate COVID-19 drug discovery.

Because the shape of the binding site of $M^{\mathrm{pro}}$ was well characterized [6,10,11,12], docking studies between ligands and the $M^{\mathrm{pro}}$ receptor have been carried out to find out possible hits or leads [13,14]. Alamri et al. used the covalent docking tool, Schrödinger’s Covalent Docking (CoVDock) [15], to discover potential covalent inhibitors from the AFCL library consisting of 1000 covalent molecules and 116 FDA-approved molecules. As a result, paritaprevir and simeprevir from FDA-approved molecules were identified as potential covalent inhibitors of $M^{\mathrm{pro}}$ of SARS-CoV-2 [13]. Peele et al. screened 24 natural plant-derived molecules with antiviral property, 22 FDA-approved antiviral drugs, and 16 antimalarial drugs with the docking approach [14]. They found several molecules, lopinavir, amodiaquine, and theaflavin digallate that showed a good binding affinity with $M^{\mathrm{pro}}$.

To further refine docking results, more rigorous absolute binding free energy calculations are performed in general. There are many approaches to obtain the absolute binding free energies of protein–ligand complexes: attach-pulling of a ligand from its receptor, linear interaction energy (LIE), molecular mechanics/Poisson–Boltzmann surface area and generalized Born surface area (MM-PBSA and MM-GBSA), free energy perturbation (FEP), thermodynamic integration (TI), and nonequilibrium molecular dynamics simulation [16,17]. All of these computational methods have a trade-off between computational cost and accuracy. Both FEP and TI approaches are considered most rigorous and accurate than the other methods. However, they demand much more computational resources to obtained well-converged binding free energies [18,19,20,21]. The ligand attach-pulling approach pulls ligand from its binding site and calculates a binding free energy based on the Jarzynski or Crooks theorem [22]. This nonequilibrium approach depends on a pulling pathway, and it is still not clear how much computational resources are required to obtain a reliable binding free energy. As an alternative approach to these perturbation methods, the MM-PB/GBSA approach, also known as an end-state method, is widely used to estimate absolute binding free energies of protein–ligand complexes due to its relatively lower computational burden. However, the approach suffers from rather large computational errors, lower accuracy, than the perturbation-based methods. Despite its lower accuracy, the MM-PB/GBSA method is becoming more popular to rank the binding free energies of different ligands with a single receptor quickly [23,24,25].

Here, we briefly review previous computational studies that used free energy calculation methods to discover possible drug candidates that bind to $M^{\mathrm{pro}}$. Ngo et al. used three computational methods, molecular docking, fast pulling of ligand (FPL), and FEP, to discovery a potential SARS-CoV-2 $M^{\mathrm{pro}}$ from the existing drugs and natural compounds [26]. They screened potential inhibitor candidates from the large library and obtained the two natural compounds that showed high binding affinity in comparison with 13b ligand, which was from the recently reported experimental work [9]. Moreover, they suggested the E166 residue of $M^{\mathrm{pro}}$ is critical in ligand binding through alanine mutation calculation. Sk et al. have evaluated the binding free energies of two molecules (α-ketoamide and Z31792168) with $M^{\mathrm{pro}}$ by using the MM-PB/GBSA approach [27]. They performed per-residue energy decomposition analysis and showed that the $\Delta E_{\mathrm{vdw}},\Delta E_{\mathrm{elec}}$, and $\Delta E_{\mathrm{nonpol}}$ terms contributed favorably to ligand binding, and the $\Delta E_{\mathrm{vdw}}$ contributed most. Based on the per-residue decomposition analysis, they identified hotspot residues, which have higher interaction energy than −1.0 kcal/mol. For both ligands, H163, H164, and M165 were identified as hot spot residues. Nutho et al. selected lopinavir and ritonavir as potential inhibitors, which were found to be effective against HIV protease [28]. From the MM-PB/GBSA analysis, they found that the $\Delta E_{\mathrm{vdw}}$ is the most favored force to binding free energy more twice than $\Delta E_{\mathrm{elec}}$ with both ligands. Moreover, they also identified important residues for ligand binding whose decomposed binding free energies are lower than −1.0 kcal/mol. Four residues, MET49, M165, P168, and Q189, were identified for the $M^{\mathrm{pro}}$-lopinavir complex and nine residues, L27, H41, M49, F140, N142, G143, H164, M165, and E166, were identified for the $M^{\mathrm{pro}}$-ritonavir complex. Wang et al. also used the MM-PBSA with a flexible docking method to identify potential inhibitors for SARS-CoV-2 $M^{\mathrm{pro}}$ protease [29]. They suggested that the five molecules, carfilzomib, ervacycline, valrubicin, lopinavir, and elbasvir, as the potential inhibitors against $M^{\mathrm{pro}}$. Among them, carfizomib was identified as the strongest binder.

The main objectives of this study are (1) to identify the hot spots of $M^{\mathrm{pro}}$ of SARS-CoV-2 via MM-PBSA calculations using multiple force fields and (2) to assess the force field dependency of MM-PBSA calculation results.

In this study, we performed the MD simulations of known 11 $M^{\mathrm{pro}}$–ligand complexes and calculated the binding affinities using MM-PBSA calculations to identify the hot spot residues of $M^{\mathrm{pro}}$. Six ligands are from Diamond Light Source (diamond.ac.uk/COVID-19/for-scientists/Main-protease-structure-and-XChem/Downloads.html), three HIV protease inhibitors (darunavir, lopinavir, and indinavir), and three more recently reported ligands (11r, 13a, and 13b) [9], respectively. For all 11 $M^{\mathrm{pro}}$–ligand complexes, we performed three MD simulations using different force fields to investigate the force field dependency of binding free energy estimates. Three different force fields were used: GROMOS54a7 (g54a7) [30,31], AMBER99SB (ff99sb) [32], and CHARMM36 force field (c36) [33]. For each complex, 100 ns of MD simulations were performed. The MM-PBSA results obtained with three force fields are compared and the hot spot residues are identified via energy decomposition analyses.

## 2. Materials and Methods

### 2.1. Molecular Docking Calculation

When we used GalaxyDock3 [34] to generate the initial $M^{\mathrm{pro}}$–ligand configurations, we selected the center of mass of the 13b (PDB ID: 6Y2F) as the center of a grid box with the SARS-CoV-2 $M^{\mathrm{pro}}$ structure whose PDB ID is 6YB7. The ligand data were downloaded from diamond.ac.uk/COVID-19. The atomic partial charges of each ligand were calculated with the Gasteiger approach [35,36]. The lowest energy docking pose was selected as the initial configuration for MD simulations of 11r, 13a, darunavir, lopinavir, and indinavir whose native structure are not identified yet.

### 2.2. Molecular Dynamics Simulations

In this study, we used two MD programs (GROMACS-2019.4 [37] and AMBER18 [38]) and three force fields: g54a7 [30,31], ff99sb [32], and c36 [33].

#### 2.2.1. MD Simulations with GROMACS

The g54a7 was used to perform MD simulations with GROMACS-2019.4 [37]. The ligand topology files were obtained from the ATB (Automated Topology Builder) server [39], which generates ligand parameters compatible with the protein force field. We used the original geometry output of ATB. The systems were solvated with the SPC water model [40] with a 12 Å padding region. We added Na^+^ ions to neutralize the system. The time step of MD simulation was set to 2 fs with the LINCS algorithm to constraint hydrogen-connected covalent bonds [41]. The long-range electrostatic interaction was calculated with the fast smooth particle-mesh Ewald electrostatics method [42,43]. The solvated system was minimized by using the steepest descent method for 10,000 steps. After minimization, a series of equilibrium MD simulations were performed: the 200 ps of NVT, 1 ns of NPT, and 50 ns of NVT with weak harmonic position restraints. At each step, the simulations were performed with the Nosé–Hoover thermostat [44] and the Parrinello–Rahman barostat [45] at 300 K. Last, the production step was performed with NPT condition during 100 ns.

#### 2.2.2. MD Simulations with AMBER

We performed MD simulations with the two force fields, ff99sb [32] and c36 [33] using AMBER18 [38].

- Preparing systems with an AMBER99SB force field (ff99sb)
  The $M^{\mathrm{pro}}$ and ligand topology files were generated using tleap [46] and Antechamber [47] programs implemented in AMBER18 with ff99sb and generalized AMBER force field (GAFF) [48]. The systems were solvated with the TIP3P water model with a padding region of 12 Å. Energy minimization calculations of the solvated systems were performed by using the steepest descent method. After energy minimization, equilibrium MD simulations were performed as follows: 200 ps of NVT, 1 ns of NPT, and 50 ns of NVT with weak harmonic positional restraints. The time step for MD simulations was set to 2 fs. To constrain all bonds involving hydrogen atoms, the SHAKE algorithm was used [49]. The particle mesh Ewald (PME) method also calculated the long-range electrostatic interactions [50]. The temperature was controlled with Langevin thermostat [51]. After equilibrium simulations were finished, we performed production simulations for 100 ns with NPT condition and the trajectory of each simulation was recorded every 2 ps.
- Preparing systems with CHARMM36 force field (c36)
  The parameter and topology files of complexes were generated from CHARMM-GUI [52] with c36 [33]. The parameters for ligands were generated by CHARMM generalized force field (CGenFF) [53,54]. The subsequent NVT 125 ps, NPT 1 ns, NVT 50 ns equilibration simulations were carried out at 300 K, followed by production runs for 100 ns under NPT ensemble condition. The TIP3P water model was used to represent explicit water molecules. We added the sodium ions in order to neutralize the system. The time step for MD simulation was set to 2 fs with the SHAKE algorithm. After the energy of the solvated system was minimized by using the steepest descent method, the subsequent steps were carried out using the identical parameters with the AMBER setting described above.

### 2.3. MM-PBSA Analysis

In this study, the interactions between the $M^{\mathrm{pro}}$ receptor and ligands were calculated with the MM-PBSA analysis. We utilized the two MM-PBSA tools; one is g_mmpbsa [55] for the GROMACS MD results and the other is MMPBSA.py [56] from the AMBER18 package, respectively.

- g_mmpbsa setting
  Each topology and parameter files required for MM-PBSA calculations are obtained from the MD simulation with GROMACS section. For a trajectory of 100 ns, 10,000 frames were selected for MM-PBSA calculations as input. For nonpoloar solvation energy calculations, we selected the SASA model [57,58,59] which is one of the most used nonpoloar models. For the other parameters, the default values of GROMACS were used.
- MMPBSA.py setting
  Each parameter and system topology files for a solvated complex, nonsolvated complex, receptor, and ligand were used from the preparation steps of MD simulation with AMBER section. Additionally, we performed the solvated $M^{\mathrm{pro}}$ receptor simulation and solvated ligand simulation, respectively, to estimate the receptor and ligand contribution in implementing MM-PBSA analysis. Similar to the g_mmpbsa setting, 10,000 frames also were used for MM-PBSA analyses with MMPBSA.py from trajectories of 100 ns. For PB calculations, the SASA (solvent accesible surface area) for the nonpoloar solvation energy used the LCPO model [60] and the ionic strength of solution was set to 0.1 M and the mbondi [61] radius set was used. The rest of the options were set to the default values of MMPBSA.py in AMBER 18 package.

### 2.4. Linear Interaction Energy (LIE) Calculation

$\Delta G_{\mathrm{LIE}}$ was computed as the mean of vdW and electrostatic interaction difference between bound state and unbound state $\Delta G_{\mathrm{LIE}}=\alpha \langle \Delta E_{\mathrm{elec}}\rangle +\beta \langle \Delta E_{\mathrm{vdW}}\rangle +\gamma$. The coefficient γ reflects the hydrophobicity of various species of inhibitors to the binding cleft conceding. The coefficient α and β are weight parameters for nonpolar and polar interactions, respectively. Based on the ligands whose experimental ${\mathrm{IC}}_{50}$ value was identified, we estimated the α, β, and γ coefficient values, followed by recalculating the other ligands’ energies to evaluate $\Delta G_{\mathrm{LIE}}$.

## 3. Results

**Figure 1:**
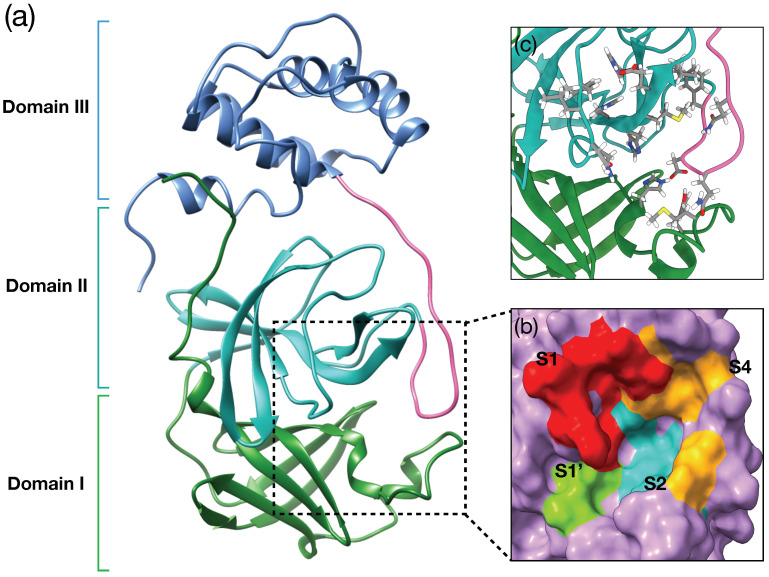
The structure of a single $M^{\mathrm{pro}}$ receptor (PDB ID: 6Y2F). (**a**) The three domains are colored and the pink colored region represents a long loop region. Domain I (green, residues 10–99), II (sea green, residues 100–184), III (cornflower blue, residues 201–305), and the pinked long loop, which is located between domains II and III (residues 185–200). The binding site is located within the dashed square box, composed of Domain I, Domain II, and the loop region. (**b**) The surface was represented by the catalytic binding center. (**c**) The surface removed view of the binding site. The residues involved in each subsite are represented.

The structure of $M^{\mathrm{pro}}$ consists of three domains: domain I (residues 10–99), domain II (residues 100–184), domain III (residues 201–303), and a long loop region (residues 185–200) (Figure 1a). Domain III is connected to domain II via the long loop structure. The substrate-binding site is located in a cleft between domain I, II with the loop region involved. It is composed of four subsites (S1, S1’, S2 and S4) (Figure 1b,c). Each subsite is spatially located and composed of consecutive amino acids in the substrate-binding site. The S1 subsite consists of F140, N142, C145, H163, E166, and H172 residues. (Figure 1b red region). The S1’ subsite is composed of T26 and L27 (Figure 1b lime region). The side chains of H41, M49, Y54, M165, D187 are involved in the formation of the S2 subsite (Figure 1b light blue). The L167, F185, Q189, Q192 consists of S4 subsite (the orange region in Figure 1b yellow).

### 3.1. MD Results of Complexes

Most MD simulations of the $M^{\mathrm{pro}}$ complexes remained bound stably, except the $M^{\mathrm{pro}}$-X0072 and -X0691 complexes. In the simulations of the $M^{\mathrm{pro}}$-X0072 complex with both ff99sb and c36, the X0072 ligand was detached from $M^{\mathrm{pro}}$. The X0691 ligand with c36 also was detached from $M^{\mathrm{pro}}$ during simulations. The trajectories showed that both the X0072 and X0691 ligands occupied a region between E166 and Q189 in S4 subsite for a while and finally were detached from $M^{\mathrm{pro}}$. These results suggest that both residues, E166 and Q189, may play the important roles of gateways of ligand binding.

The spatial occupancy patterns of ligands sampled during simulations are visually investigated to identify which residues participate in ligand binding of $M^{\mathrm{pro}}$. To perform this investigation, the ’grid’ command of the CPPTRAJ [62] program of the AMBER18 package was used to bin the distribution of a ligand into a 3D grid. Before performing the analyses, periodic boundary conditions were removed and centered the system to put a ligand at the origin of each trajectory.

**Figure 2:**
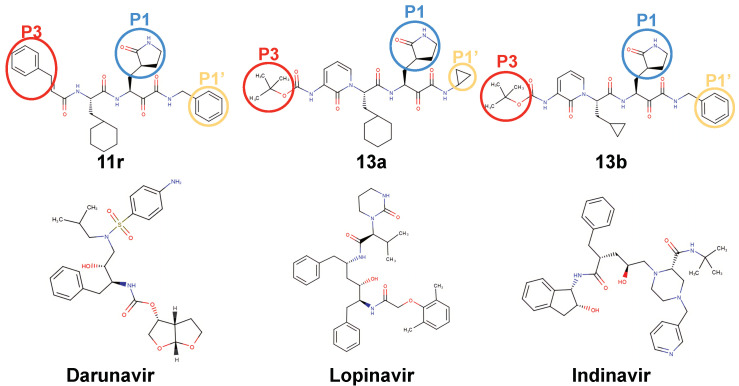
The ligands analyzed in Results section. 11r, 13a, and 13b ligands are at the upper row. The branches of 11r, 13a, and 13b ligands were represented in colored-circle. Darunavir, lopinavir, and indinavir are in the lower row. All ligands anaylzed in this study are in Figures S1–S5.

**Figure 3:**
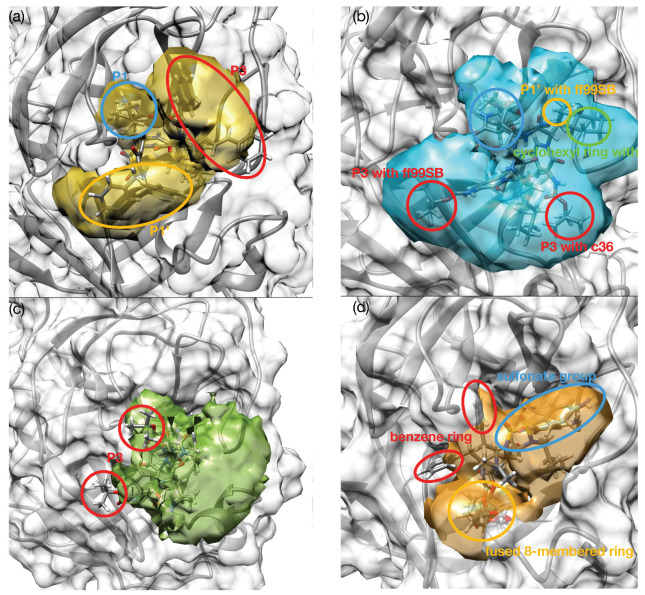
The examples of 3D histograms of ligand occupancy, comparing MS simulation results using ff99sb and c36. The colored region is a 3D histogram. The 3D histogram is spatial distribution of ligand atom sampled during simulation with ff99sb. The representative conformations from both ff99sb and c36 are imposed. (**a**) 11r (**b**) 13a (**c**) 13b (**d**) darunavir ligand.

The MD result of darunavir showed a significantly localized distribution than those of lopinavir and indinavir, which are the inhibitors of HIV protease. The sulfonate group of darunavir stayed at the S4 subsite and the benzene ring also was located in S1 subsite and did not fluctuate much (Figure 2 and Figure 3d). However, in the case of lopinavir, the functional groups of lopinavir fluctuated much, except that the dimethyl substituted benzene group stayed at the S4 subsite. The branches of indinavir showed that the rings fluctuated, and did not occupy the S4 subsite similar to other HIV protease inhibitors. The other ligands, 11r and 13a showed different binding behaviors from 13b. The ligands, 11r and 13a, showed more concentrated spatial distribution consistently than the 13b ligand in simulations performed with all force fields. On the contrary, the trajectory of 13b showed more widely distributed 3D histograms because of the fluctuation of its P1, P3 branches (Figure 2 and Figure 3a–c). The P1 branch of both 11r and 13a remained stably at the S1 subsite during simulations. The P3 branch of 11r and the P1’ part of 13a with ff99sb, and the cyclohexyl ring of 13a with c36 also remained steadily at the S4 subsite, where most strong binders are occupied significantly during simulations. However, the P1 branch of 13b was located at a different region compared with 11r and 13a and fluctuated more than them, and 13b did not occupy the S4 subsite between E166 and Q189 during the simulations. It appears that this dynamical behaviors of the ligands are consistent with experimental data, the stronger binding free energy of 13b than 11r and 13a, which will be discussed further in the next section.

The fact that the S4 subsite was occupied by many strong binders was employed in a previous study that developed a new drug candidate from a weak hit molecule, perampanel [63]. The authors performed a structure-based substrate scope analysis, which considers the interaction and environments between $M^{\mathrm{pro}}$ and hit molecules, and rationally changed the branches of perampanel to fill the void of the S4 subsite. Most modified ligands interacted strongly with the E166, Q189, and Q192 residues. Especially, most modified ligands whose chloride atom was substituted for various alkoxy groups such as propoxy, butoxy group, or more bulky benzyloxy group to occupy the S4 subsite region, became more than 10 times stronger than the hit molecule in terms of ${\mathrm{IC}}_{50}$ values. This tendency is consistent with the MM-PBSA results, which will be discussed in the next section and may be attributed to hydrogen bonds between ligands and $M^{\mathrm{pro}}$. The complete results of all the 3D histogram results are illustrated in Supplementary Information (Figures S15–S17).

### 3.2. Comparison of MD Simulation Results of Complexes Using Different Force Fields

**Figure 4:**
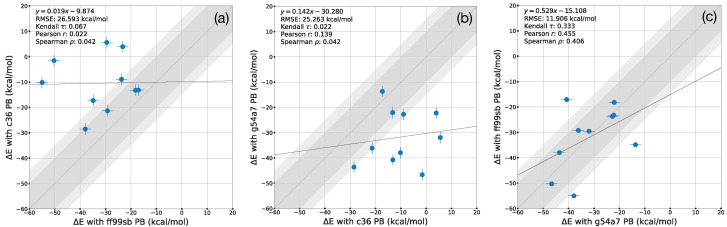
A comparison of the $\Delta E_{\mathrm{MM}}$ obtained with MM-PBSA calculations using ff99sb, c36, and g54a7. (**a**) A comparison of MM-PBSA results between ff99sb and c36 (**b**) A comparison of MM-PBSA results between c36 and g54a7 (**c**) A comparison of MM-PBSA results between g54a7 and ff99sb The dashed diagonal line corresponds to the perfect agreement between the two results. The dotted lines correspond to a range of an absolute error of 10 kcal/mol. The error bars are presented with points. Because the X0072 and X0691 ligand were detached from $M^{\mathrm{pro}}$, they were omitted.

In this section, we compared the MM-PBSA calculation results with three different force fields to investigate the effect of force field parameters on binding free energy estimates (Figure 4 and Figures S6–S11). We estimated the entropic contribution of each ligand’s binding free energy by using normal mode analysis (Table 1). The estimated entropy values were similar in the range of ±1 kcal/mol. Thus, entropic contributions are not considered for analysis.

#### 3.2.1. A Comparison of ff99sb and c36 Force Field Results

The MM-PBSA results obtained with ff99sb and c36 showed weak correlations between them, Kendall τ=0.067, Pearson r = 0.022, and Spearman ρ=0.042, respectively (Figure 4a). Overall, the MM-PBSA binding free energy estimates obtained with ff99sb were more favorable than those with c36 simulations. Most binding energy results obtained with MM-PBSA calculations using ff99sb had lower bind energy by more than about 10 kcal/mol than the c36 results.

The strongest binders of each force field are 11r (−54.990 kcal/mol) with ff99sb and 13a (−28.523 kcal/mol) with c36, respectively. 13a also identified as the top3 strong binder with ff99sb (−37.971 kcal/mol). However, 11r was not identified as a strong binder with c36. It suggested that the two force fields did show consistent results with each other. From experiments [9,64], the binding free energies of 11r, 13a, 13b, and darunavir ligands were −9.23, −7.70, −8.45, and −6.14 kcal/mol, respectively. In terms of consistency with experimental data, MM-PBSA with ff99sb correctly identified 11r as the strongest binder. However, c36 results predicted that 13a would be a stronger binder than 11r, which is inconsistent with the experiment. Both ff99sb (−29.535 kcal/mol) and c36 (5.579 kcal/mol) estimated that 13b is the weakest binder among the 11r, 13a, and 13b despite 13b is the second strong binder based on the experiment. We assumed the fact that 13b did not occupy the S4 subsite unlike 11r and 13a may explain why the MM-PBSA results were not consistent with the experiment among 11r, 13a, and 13b ligands. Moreover, although darunavir is the weakest binder from the experimental data, the two force field failed to estimate the fact that darunavir is the weakest binder.

#### 3.2.2. A Comparison of c36 and g54a7 Force Field Results

Next, we compared the MM-PBSA results obtained with c36 and g54a7 (Figure 4b). A weak correlation was also observed with a correlation coefficient, Kendall τ=0.022, Pearson *r* = 0.139, and Spearman ρ=0.042, respectively. Most binding energies obtained with g54a7 showed more favorable values than those of c36. MM-PBSA results with g54a7 estimated that darunavir would be the strongest binder with a binding energy of −46.640 kcal/mol. The top contributing residues for the binding of darunavir with g54a7 were M49, E166, M165, and Q189 with decomposed energies (−3.625, −2.740, −1.972, and −1.491 kcal/mol, respectively). Because the M165, E166, and Q189 residues are in the S4 subsite, g54a7 also suggested that the S4 subsite was important for $M^{\mathrm{pro}}$-ligand binding. Although c36 showed less favorable energies than g54a7, it is valuable to compare the binding energy of darunavir, which is the strongest binding with g54a7, between g54a7 and c36. With c36, both E166 and Q189 had the unfavorable decomposed energies—4.748 and 1.314 kcal/mol, respectively. However, these energies were reversed with g54a7—−2.740 and −1.491, respectively. We found that both residues have with highly unfavorable electrostatic interactions with the ligand, E_elec_ of 19.815, 4.431 kcal/mol, respectively.

#### 3.2.3. A Comparison of g54a7 and ff99sb Force Field Results

The MM-PBSA binding energies obtained with ff99sb and g54a7 were relatively well-correlated with Kendall τ=0.333, Pearson *r* = 0.455, and Spearman ρ=0.406, respectively (Figure 4a,c). g54a7 also estimated that 13b is the weakest binder among 11r, 13a, 13b, and darunavir. These results were consistent with all three force fields. The MM-PBSA results with ff99sb showed a similar trend with the MM-PBSA results with g54a7 except for 11r and lopinavir cases. If the three outliers are excluded, a correlation coefficient between g54a7 and ff99sb was ρ=0.933. This result suggests that g54a7 yields similar binding free energies with the all-atom model from ff99sb, although g54a7 used united-atom model parameters. The strongest binder without the outlier ligand was darunavir: −50.288 kcal/mol with ff99sb and −46.640 kcal/mol with g54a7. Darunavir was also the second strongest binder with the outlier ligands with ff99sb.

**Figure 5:**
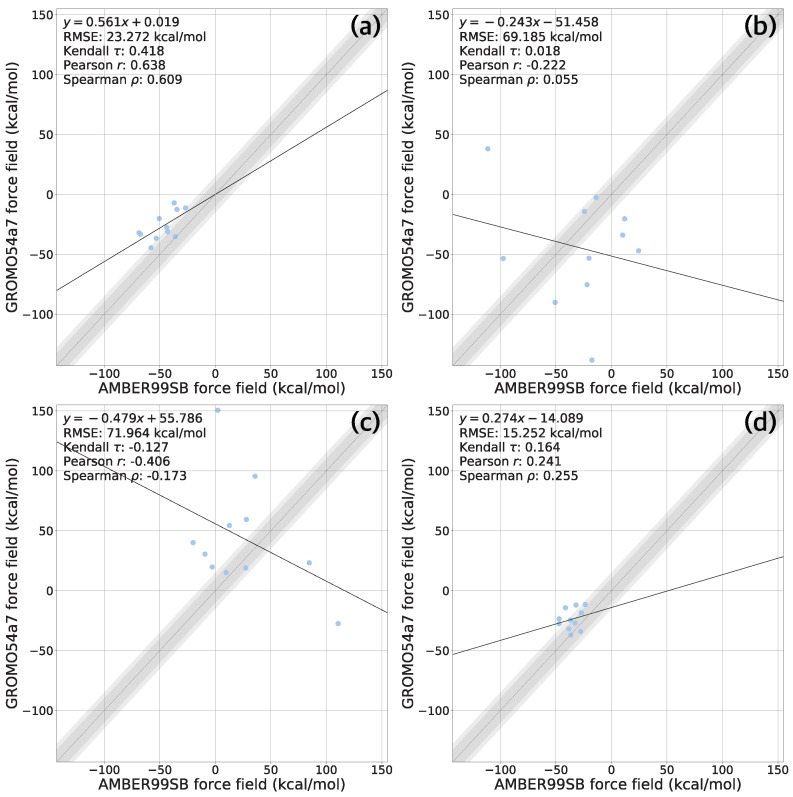
A comparison of the ΔE obtained with MM-PBSA calculations between ff99sb and g54a7. (**a**) $\Delta E_{\mathrm{vdw}}$, (**b**) $\Delta E_{\mathrm{elec}}$, (**c**) $\Delta E_{\mathrm{PB}}$, and (**d**) $\Delta E_{\mathrm{nonpol}}$ All the subfigures are same in *x*- and *y*-axis scale to compare easily. The dashed diagonal line corresponds to the perfect agreement between the two results. The dotted lines correspond to a range of an absolute error of 10 kcal/mol. Because the X0072 and X0691 ligand were detached from $M^{\mathrm{pro}}$, they were omitted.

To investigate why the results of ff99sb and g54a7 are correlated, we calculated the correlation coefficients between individual energy terms of MM-PBSA results: $\Delta E_{\mathrm{vdw}}$, $\Delta E_{\mathrm{elec}}$, $\Delta E_{\mathrm{PB}}$, and $\Delta E_{\mathrm{nonpol}}$, respectively (Figure 5). Among the energy terms, the $\Delta E_{\mathrm{vdw}}$ term showed a correlation between two force fields while the other energetic terms did not show any correlation. This shows that a correlation between ff99sb and g54a7 can be attributed to the van der Waals term. This may be due to the fact that electrostatic or solvation interaction terms depend on more delicate parameters, such as partial charges and atomic radii for Poisson–Boltzmann calculations

### 3.3. Linear Interaction Energy Analysis

**Figure 6:**
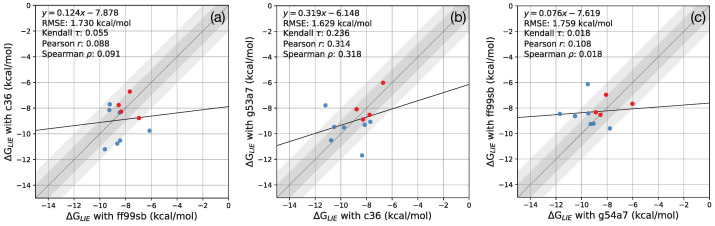
A comparison between MM-PBSA calculation results fitted by the linear interaction energy analysis. The left plot corresponds to the correlation between c36 and ff99sb. (**a**) A comparison between ff99sb and c36 results fitted by the LIE analysis. (**b**) A comparison between c36 and g54a7 results fitted by the LIE analysis. (**c**) A comparison between g54a7 and ff99sb results fitted by the LIE analysis. The red dots correspond to the ligand whose experimental ${\mathrm{IC}}_{50}$ values are identified. The dark shaded and light shaded regions correspond to an absolute error of 1 and 2 kcal/mol, respectively.

To improve the accuracy of binding affinity calculations, LIE analysis is commonly used to tune the balance between the electrostatic and van der Waals interactions. To check whether the LIE analysis improves the correlations between the calculated results and experiments, we performed the LIE analysis using the four ligands whose experimental ${\mathrm{IC}}_{50}$ were known. (Figure 6).

It is identified that the LIE analysis did not improve the correlations between results obtained with different force fields. The correlation coefficient between the results of ff99sb and g54a7 deteriorated from 0.45 to 0.11. Meanwhile, the correlation coefficient between c36 and g54a7 elevated to 0.31 from 0.14, which is the highest among the LIE results. The correlation between c36 and ff99sb remained low after the LIE analysis with a Pearson correlation coefficient of 0.09. These results demonstrate that, even with the LIE analysis, the MM-PBSA results strongly depend on the choice of force field.

### 3.4. A Comparison of Polar Solvation Energy

**Figure 7:**
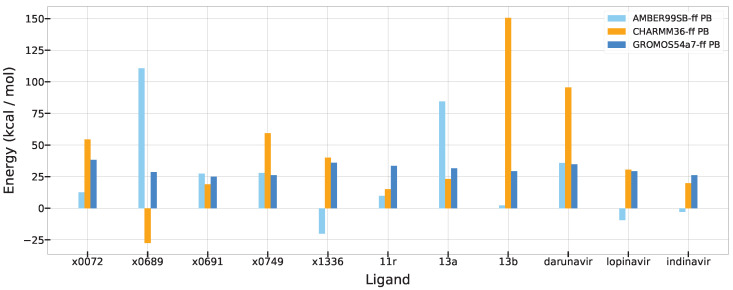
A comparison of $\Delta E_{\mathrm{PB}}$ with ff99, c36, and g54a7 force fields from MM-PBSA analysis.

In this section, we compared $\Delta E_{\mathrm{PB}}$ from the MM-PBSA calculation results with three different force fields to investigate the effect of solvation on ligand binding shown in Figure 7. Usually, the PB energies contributed unfavorably to ligand binding. Specifically, the c36 force field showed the most unfavorable contribution for the binding of 13b and darunavir. 13b with c36 showed that E166 and R188 have $\Delta E_{\mathrm{PB}}$ of 19.1 and 15.3 kcal/mol, respectively. Moreover, darunavir with c36 also showed that E166 and R188 have $\Delta E_{\mathrm{PB}}$ of 19.8 and 11.3 kcal/mol, respectively, while 13a contributed to $\Delta E_{\mathrm{PB}}$ of 6.2 and 4.9 kcal/mol for E166 and R188, respectively.

On the other hand, in the cases of lopinavir and indinavir, which are moderate binders with ff99sb, the $\Delta E_{\mathrm{PB}}$ showed favorable energy. The exposed residues showed highly favorable $\Delta E_{\mathrm{PB}}$. The D263 residue had highly favorable $\Delta E_{\mathrm{PB}}$ of −20.6 kcal/mol with lopinavir and −19.7 kcal/mol with indinavir, respectively, whereas that of 11r is only −5.6 kcal/mol. Additionally, E178 also showed highly favorable $\Delta E_{\mathrm{PB}}$ of −16.6 kcal/mol with lopinavir and −19.0 kcal/mol with indinavir, while that of 11r is only −11.3 kcal/mol.

### 3.5. Summary of MM-PBSA Results

**Figure 8:**
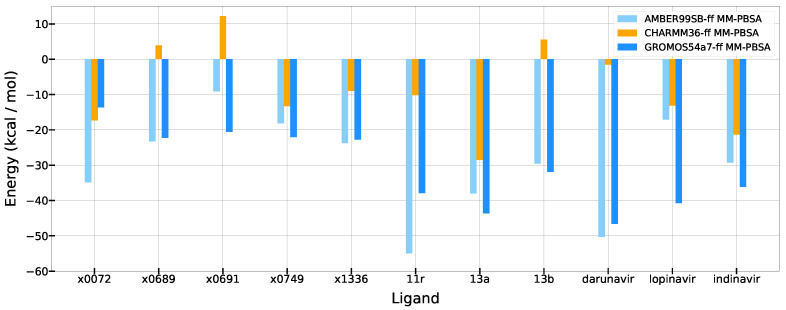
Overall binding free energy from MM-PBSA. All values has kcal/mol energy unit.

In summary, the MM-PBSA estimates obtained with c36 is showing a different trend with those obtained with ff99sb and g54a7 (Table 1 and Figure 8). The absolute values of binding energies obtained with c36 are smaller than those with ff99sb and g54a7. Unfortunately, all three force fields failed to predict the rankings of known experimental binding affinity data. All three force fields predicted that 13b ligand was the weakest binder among 11r, 13a, and 13b; however, 13b was the second strong binder based on the experimental data. Only ff99sb correctly identified the 11r ligand as the strongest binder. However, more experimental data are necessary to evaluate the accuracy of force fields more rigorously in obtaining correct absolute binding free energies of $M^{\mathrm{pro}}$ complexes.

These results show that, based on the MM-PBSA calculations, no force field is accurate enough to obtain the absolute binding affinities of $M^{\mathrm{pro}}$-ligand complexes yet, and the calculation results may show large variations according to the tested force field. Thus, our results also show that a care should be taken to interpret the absolute binding free energy estimates obtained with MM-PBSA and different force fields. These results also emphasizes the necessity of using more rigorous absolute binding free energies approaches [65,66,67,68]. The complete results of all energies are Supplementary Information Table S1.

### 3.6. Hot Spot Residues for Ligand Binding

**Figure 9:**
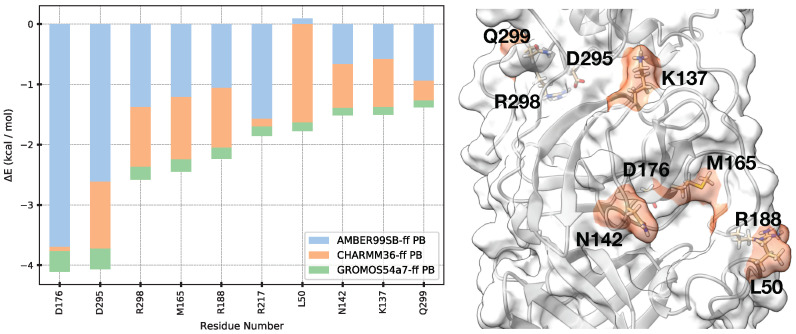
Averaged contribution energy from MM-PBSA and corresponding hot spot residues (**left**) are illustrated. Hot spot residues are highlighted on the surface of $M^{\mathrm{pro}}$ (**right**). Because the X0072 and X0691 ligands were detached from $M^{\mathrm{pro}}$, they were omitted when generating the figure. Because the terminal residues are in the backside of the figure, their orange-colored regions were hidden.

To identify hot spot residues playing important roles in ligand binding, we performed a per-residue energy decomposition analysis and calculated the average binding energy contribution of all ligands. The top 10 residues that contribute most to ligand binding of $M^{\mathrm{pro}}$ based on the average per-residue interaction energy is illustrated in Figure 9. All the top 10 contributing residues have $\Delta E^{\mathrm{MM}}$ lower than −1.0 kcal/mol on average.

The M165 residue is identified as the top contributing residue consistently across all tested force fields. M165 showed $\Delta E_{\mathrm{avg}}^{\mathrm{contrib}}=-5.0\mathrm{kcal}/\mathrm{mol}$ which is 2∼3 fold stronger than other residues’ $\Delta E^{\mathrm{MM}}$. This result is consistent with the fact that most strongly binding ligands remained stably around the S4 subsite. The decomposition analysis revealed that M165 interacts favorably with ligands via both electrostatic and van der Waals interactions. With the strongest binder 11r with ff99sb and 13a with c36, M165 also was the most favorable residue. For the 11r case, $E_{\mathrm{vdw}}$, $E_{\mathrm{elec}}$, and $E_{\mathrm{PB}}$ were −2.508, −1.977, and 1.462 kcal/mol with ff99sb and −1.564, −1.614, and 1.714 kcal/mol with c36, respectively. For the 13a case, $E_{\mathrm{vdw}}$, $E_{\mathrm{elec}}$, and $E_{\mathrm{PB}}$ were −1.304, 0.842, and 1.003 kcal/mol with ff99sb and −1.721, −0.910, and 1.343 kcal/mol with c36, respectively. These results show that $E_{\mathrm{vdw}}$ mostly contribute to ligand binding.

From the atomic contact figures (Figures S18 and S19) generated by ligPlot+ [69], we identified that there are many short-range interactions between $M^{\mathrm{pro}}$ and the ligands, represented by red semicircles on protein residues or ligand atoms. Many atoms in the rings of ligands, mostly aromatic rings, are in close contact with the binding site residues. For example, most nonpolar atoms of three six-membered rings (two benzene rings and one cyclohexyl ring) of 11r showed that they form close contacts with binding site residues: S46 with the P1’ benzene ring, E166 with the P3 benzene ring, and H164, M165, R188, and Q189 with the cyclohexyl ring.

Additionally, indinavir, 13a, and 13b have a large nonpolar group, a *tert*-butyl group, participating in protein–ligand binding (Figure S18). The *tert*-butyl groups of the ligands have many close contacts with surrounding residues: N119 with 13a, and N119 with 13b, and L141, S144, C145, and H164 with indinavir. Specifically, N119, which is away from the center of the binding site, largely interacts with ligands. Thus, we assume that the *tert*-butyl groups interact strongly with N119 via van der Waals interaction due to its bulky volume. Therefore, nonpolar groups, such as ring structure and *tert*-butyl group, play important roles in $M^{\mathrm{pro}}$–ligand binding with many residues at diverse locations. The presence of many contacts between nonpolar atoms makes $E_{\mathrm{vdw}}$ the most contributing energetic term than the other terms.

Based on the fact that 11r with ff99sb and 13a with c36 are the strongest binders, we performed a residuewise binding free energy decomposition analysis to identify the hot spot residues of $M^{\mathrm{pro}}$. The top contributing residues of 11r with ff99sb were D295, D187, D176, and H163 with binding energies of −5.538, −4.983, −4.350 and −3.624 kcal/mol, respectively. Except for D176 (4.185 kcal/mol), their $E_{\mathrm{elec}}$ were lower than −5.0 kcal/mol. The top contribution residues of 13a with c36 were D295, Y161, R298, L58, L50 with binding energies of −3.457, −3.308, −3.195, and −3.025 kcal/mol, respectively. They also had low $E_{\mathrm{elec}}$ except for Y161 (1.435 kcal/mol).

Interestingly, the terminal charged residues, D295, R298, and Q299, which are far from the binding site interacted strongly with ligands. Although they were far from the binding site over about 30 Å during MD simulation, the energy decomposition analysis showed that these residues have strong electrostatic or polar solvation attractive interaction for ligand’s binding. Recently, T. Sztain et al. reported that the C-terminal region was examined as a potential allosteric site [70], which is consistent with our per-residue decomposition analysis result.

It is observed that the residues consisting of the S1’ subsite contributed less to ligand binding than the other subsites, which is consistent with the 3D histogram analysis. Except for the terminal D295, R298, and Q299 residues that are far from the binding site, the hot spot residues (N142, M165, D176 and, R188) are identified in the S1 or S4 subsites. This result suggests that the residues of the S4 and S1 subsites may interact stronger than the residues of the S1’ and S2 subsites with ligands.

### 3.7. Hydrogen Bonds Analysis

**Figure 10:**
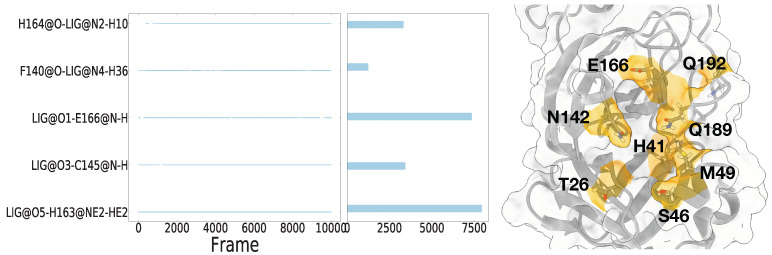
The hydrogen bond analysis of 11r, which was strongest binder with ff99sb at left. The residues that were frequently captured in hydrogen bond analysis at right.

We carried out a hydrogen bond analysis and investigated its percentage to find which residues are forming hydrogen bonds strongly with the ligands. The complete hydrogen bond analysis results are in Supplementary Information Figures S12–S14. It is identified that if a specific hydrogen bond lasts long during a simulation, it contributed strongly to binding free energy. For the cases of 11r and 13a, which were the strongest binders with ff99sb and c36, respectively, the hydrogen bonds that existed for more than 60% of the entire trajectory were identified to be the most strongly contributing residues H142, S144, 163, and E166. Noticeably, the 11r ligand with ff99sb formed two stable hydrogen bonds, which existed more than 70% of the entire trajectory (Figure 10).

For the simulation of 11r with ff99sb, the hydrogen atom attached at the NE atom of H163 formed hydrogen bonds with 11r for 79% of the trajectory. The hydrogen atom at the N atom of E166 formed hydrogen bonds with 11r for 73%. For the simulation of darunavir with ff99sb, the oxygen atom at H164 and the hydrogen atom at the N atom of E166 also formed the hydrogen bonds with darunavir with 84% and 66%, respectively. For the case of 13a with c36, which is the strongest binder with c36, the hydrogen atom at N atom of E166 also existed for 61%, while other hydrogen bonds existed lower than 10% of the trajectory. Except for the ligands discussed above (11r, 13a, and darunavir), the other ligands showed that the $M^{\mathrm{pro}}$–ligand complexes formed hydrogen bonds under 30% with both ff99sb and c36 (Table 2). For example, X0749, which is the weakest binder with ff99sb, showed that all hydrogen bonds lasted lower than 10%. These results show that hydrogen bonds play important roles in strong binding affinity between $M^{\mathrm{pro}}$–ligand complexes.

The residues consisting of the subsites formed diverse hydrogen bonds during MD simulation with all of ligands (Table 3 and Table 4). N142, E166, and Q189 formed highly stable hydrogen bonds with the ligands. The atoms that were frequently involved in forming hydrogen bonds were similar both with ff99sb and c36. Based on these results, the stable hydrogen bonds appeared to be independent of the force field. Most stable hydrogen bonds were formed by the atoms in side chain. Except for the H-N atom in E166, all the frequencies of each backbone atom involved in hydrogen bonds were less than 10 times. This section may be divided by subheadings. It should provide a concise and precise description of the experimental results, their interpretation, and the experimental conclusions that can be drawn.

## 4. Discussion

The ligand binding behaviors depended on which functional group (named branch in our manuscript) is interacting with the binding site and how a ligand posed in the binding site. Thus, we investigated their binding behaviors where the branches of ligands were located at the binding site and how they fluctuated during the MD simulations. In terms of binding pose, the 11r, 13a, and 13b ligands’ branches are located at different subsites for 11r, 13a, and 13b (Figure 3, Figures S15 and S16).

First, the P1 branch, which is a five-membered ring, is a common functional group for 11r, 13a, and 13b. The P1 branch of 11r and 13a consistently occupied the S1 subsite with both ff99sb and c36 (blue circle in Figure 3a,b). With the ff99sb force field, the RMSF values of the P1 branch of 11r and 13a are 11.1 Å and 10.7 Å, respectively. However, the P1 of 13b did not occupy the S1 subsite with an RMSF value of 14.9 Å. These results suggest that the occupancy of the S1 subsite of the P1 branch may play an important role in $M^{\mathrm{pro}}$–ligand binding.

Second, the P3 branch of 13a (red circle in Figure 3b, RMSF; 15.9 Å with ff99sb and 14.6 Å with c36, respectively) fluctuated more than the P1’ branch of 11r (yellow circle in Figure 3a, RMSF; 13.2 Å with ff99sb and 15.4 Å with c36, respectively) at the S1’ subsite.

Additionally, the exposed P3 branch of 13b (red circle in Figure 3c) fluctuated most during simulations in terms of RMSF of 18.6 Å with ff99sb and 15.2 Å with c36, respectively. Although the different branch are located at the S1’ subsite, the RMSF values indicate that occupancy of the S1’ subsite is less important in ligand binding.

Furthermore, hydrogen bond patterns between the ligands and the binding site residues also showed different patterns by ligands. With ff99sb, 13b formed hydrogen bonds with $M^{\mathrm{pro}}$ less frequently than 11r and 13a (Figure S12). 11r formed hydrogen bonds with H163 and E166 for more than 75% of the trajectory (Figure S12f). 13a formed hydrogen bonds with N142 and S144 for more than 40% (Figure S12g). However, 13b did not form any hydrogen bond with a frequency of more than 40% (Figure S12h). The maximum frequency of hydrogen bond of 13b is about 20%, which is less than the halves of those of 11r and 13a. These indicate that 11r and 13a stay longer in a specific binding pose or location than 13b because the corresponding binding pose is more stable than the others. This pattern is also consistent with the MM-PBSA results; ΔG of 11r and 13a are lower than that of 13b with ff99sb in Figure 8.

In addition to the hydrogen bonds mentioned above, most other hydrogen bonds of 11r and 13a formed for more than 20 % of the trajectory, whereas 13b did not. This suggests that 11r and 13a strongly interact with residues at the S1 (F140, N142, C145, H163, E166, and H172) and the S4 (L167, F185, Q189, Q192) subsite (Figure S12f,g). Thus, this supports the fact that MM-PBSA analysis can identify the hot spot residues discussed in Section 3.6. Moreover, only 13b formed hydrogen bonds with the S46 and the T26 residues, which were not observed in the hydrogen bond patterns of 11r and 13a. T26 and S46 residues are located at the S1’ and S2 subsites, respectively. It suggests that the 13b can more interact with residues in S1’ andS2 subsite, resulting in different ligand behaviors compared to 11r and 13a. This result is also consistent with the c36 force field results. 13a, the strongest binder with c36, showed the most stable hydrogen bond among 11r, 13a, and 13b. Furthermore, 13b more frequently formed a hydrogen bond with T26 in Figure S13.

In this study, we utilized the MM-PBSA approach to investigate the hot spot residues of SARS-CoV-2 $M^{\mathrm{pro}}$ with three different force fields: ff99sb, c36, and g54a7. There was no study that performed a comparison between different force fields. Here, we used the force fields ff99sb, c36, and g54a7 and compared their MM-PBSA analysis results. If the MM-PBSA methodology and all three force fields are well matured, all binding affinity estimates would have been be similar and show a reasonable correlation.

However, the estimated absolute binding energies of $M^{\mathrm{pro}}$ and known ligands calculated with MM-PBSA were not consistent between three different force fields. By comparing the MM-PBSA results from the tested force fields, we found that only the ff99sb and g54a7 results have a weak correlation. On the contrary, the c36 results showed almost no correlation with the g54a7 and ff99sb results. The binding energies from c36 were consistently estimated higher than those of both ff99sb and g54a7. These results suggest that MM-PBSA results heavily depend on the choice of force field and that no single force field is able to predict the correct relative ranking of experimental binding affinities of the $M^{\mathrm{pro}}$ complexes. In other words, one should be careful in comparing binding free energy estimates obtained with different force fields and the MM-PBSA method, suggesting that the accuracy of MM-PBSA calculation is limited. From a practical perspective of drug design, our results indicate that the MM-PBSA analysis should be used only in the early stage of drug discovery and more rigorous absolute binding free energy methods, such as alchemical relative/absolute binding free energy calculations [26,71], should be used in later stages for more accurate lead optimization.

Based on the residuewise decomposed energies, the terminal residues (D295, R298, and Q299) were identified to contribute strongly to ligand binding. Because they were far from the known binding site, they were not considered as a residue that contributes the binding energy at first glance. However, the terminal residues had averaged decomposed energy in our ligands: −4.065, −2.582, and −1.383 kcal/mol, respectively, which has been suggested by the other study that the terminal residues were important in ligand binding [70]. The decomposed residue energies showed that the residues of the S1 or S4 subsites contributed more strongly to binding energy than the residues in the S1’ or S2 subsites, supporting that the ligand’s atoms or branches located in S1’s subsite fluctuated more freely than those residues in the S1 or S4 subsites.

## 5. Conclusions

In growing threat of COVID-19, SARS-CoV-2 main protease ($M^{\mathrm{pro}}$) is one of the potential pharmaceutical target protein because $M^{\mathrm{pro}}$ enzyme has no human analogue and is conserved among corona viruses [5].

There have been computational studies to predict the binding affinities of $M^{\mathrm{pro}}$–ligand complexes [13,28,29]. Previous studies only performed MM-GBSA [13,29] or investigated only two ligands [28], using only one force field, which may affect the prediction quality heavily. Compared to previous computational studies, we performed MD simulations of 11 $M^{\mathrm{pro}}$–ligand complexes with three different force fields and followed by the MM-PBSA analysis, which yields more accurate binding free energy estimates in general than MM-GBSA. There was no study that performed a comparison between different force fields. Here, we used the force fields ff99sb, c36, and g54a7 and compared their MM-PBSA analysis results. If the MM-PBSA methodology and all three force fields are well matured, all binding affinity estimates would have been be similar and show a reasonable correlation.

In this study, we investigated the binding affinity of SARS-CoV-2 $M^{\mathrm{pro}}$ with its potential inhibitors to offer insight about $M^{\mathrm{pro}}$–ligand binding and accelerate $M^{\mathrm{pro}}$ drug discovery. We benchmarked the absolute binding free energy estimated obtained with three widely used force fields, ff99sb, c36, and g54a7. Unexpectedly, we identified that MM-PBSA results with different force fields are not consistent with each other. Only the results with ff99sb and g54a7 are weakly correlated. These results suggests that MM-PBSA results highly depend on the choice of force field. This also shows that one should be careful in comparing binding free energy estimates of $M^{\mathrm{pro}}$–ligand complexes obtained with different force fields and the MM-PBSA method. Through energy decomposition analysis, we found that the residues of $M^{\mathrm{pro}}$ at the S1, S4 subsites or terminal regions play essential roles in ligand binding. This suggests that these residues may play important roles in developing drugs. We hope that our results could provide insight into the design and development of more potency inhibitor against SARS-CoV-2 $M^{\mathrm{pro}}$ and facilitate the drug development for the treatment of COVID-19.

## Figures and Tables

**Table 1:** Overall MM-PBSA difference energy results. All values in a kcal/mol energy unit. The superscript ^*a*^ means that all the energies are from the AMBER18 MD engine with ff99sb, while the superscript ^*b*^ indicates the results from the AMBER18 MD engine with c36. The superscript ^*c*^ indicates that all the energy values are from GROMACS-2019.4 MD engine with g54a7. NMA ΔS is that the entropy is calculated with normal mode analysis and QH ΔS means that the entropy is estimated by quasiharmonic (QH) approximation with only c36 system because MMPBSA.py package only supports normal mode analysis with ff99sb, not c36. All entropy values are computed by MMPBSA.py in AMBER18 MD package.

Ligand	MM-PBSA ^*a*^	MM-PBSA ^*b*^	MM-PBSA ^*c*^	NMA ΔS	QH ΔS
X0072	−34.857	−17.302	−13.659	−16.455	−12.441
X0689	−23.223	3.943	−22.241	−16.899	−12.723
X0691	−9.075	12.182	−20.549	−18.119	−12.761
X0749	−18.167	−13.242	−22.040	−17.327	−12.677
X1336	−23.727	−8.932	−22.734	−19.280	−12.791
11r	−54.990	−10.177	−37.905	−31.088	−13.369
13a	−37.971	−28.523	−43.613	−27.803	−13.389
13b	−29.535	5.579	−31.927	−25.499	−13.404
darunavir	−50.288	−1.556	−46.640	−29.247	−13.330
indinavir	−29.238	−21.343	−36.142	−30.431	−13.430
lopinavir	−17.064	−13.166	−40.744	−27.693	−13.451

**Table 2:** The number of hydrogen bonds of each ligand, which satisfies the thresholds with both ff99sb and c36. The total 10,000 number of frames was used to perform hydrogen bond analysis.

Threshold	0.1		0.2		0.3		0.5		0.7	
**Ligand**	**ff99sb**	**c36**	**ff99sb**	**c36**	**ff99sb**	**c36**	**ff99sb**	**c36**	**ff99sb**	**c36**
X0072	0	0	0	0	0	0	0	0	0	0
X0689	2	2	1	2	1	1	0	1	0	1
X0691	1	0	0	0	0	0	0	0	0	0
X0749	0	0	0	0	0	0	0	0	0	0
X1336	3	1	1	0	0	0	0	0	0	0
11r	7	2	4	1	4	1	2	0	2	0
13a	6	1	6	1	2	1	0	1	0	0
13b	2	3	0	1	0	1	0	0	0	0
darunavir	4	4	3	3	3	2	3	1	1	0
lopinavir	5	2	1	1	1	0	0	0	0	0
indinavir	3	1	2	1	1	0	1	0	0	0

**Table 3:** The $M^{\mathrm{pro}}$ backbone atoms on residues that frequently formed hydrogen bonds with ff99sb and c36 during simulations. The atoms were based on the hydrogen bonds in the top 5 existence percentage of each ligand MD simulation by hydrogen bond analysis. The number in parentheses indicates how many atoms formed hydrogen bonds in all ligand cases.

Residue	Backbone	
**Force Field**	**ff99sb**	**c36**
T26	H-N(5), O(2)	H-N(7)
H41	O(1)	O(1)
S46	O(3), H-N(5)	O(1), H-N(5)
N142	H-N(1), O(1)	H-N(1)
E166	H-N(10), O(4)	H-N(7)
Q189	O(3), H-N(2)	O(3), H-N(1)

**Table 4:** The $M^{\mathrm{pro}}$ side chain atoms on residues that frequently formed hydrogen bonds with ff99sb and c36 during simulations. The atoms were based on the hydrogen bonds in the top 5 existence percentage of each ligand MD simulation by hydrogen bond analysis. The number in parentheses means that how many atoms formed hydrogen bonds in all ligand cases.

Residue	Side Chain	
**Force Field**	**ff99sb**	**c36**
T26	HG1-OG1(2), OG1(1)	HG1-OG1(3)
H41	HE2-NE2(7), ND1(2), NE2(1)	HD1-ND1(5), NE2(2)
S46	HG-OG(10), OG(7)	HG-OG(7), OG(4)
N142	HD21-ND2(10), HD22-ND2(8),	HD22-ND2(8), HD21-ND2(7),
	OD1(12), ND2(3)	OD1(6), ND2(3)
E166	OE1(6), OE2(7)	OE1(6), OE2(4)
Q189	HE22-NE2(10), HE21-NE2(10), OE1(1), NE2(1)	HE21-NE2(11), HE22-NE2(11), OE1(15), NE2(1)

## Data Availability

Not applicable.

## Supplementary Materials

The following are available online at https://www.mdpi.com/article/10.3390/life12010054/s1, Figure S1: The used ligands in this work. Figure S2: The partial charge represented ligand figures generated by gaff. Ligands are x0072, x0689, x0691, x0749, and x1336. Figure S3: The partial charge represented ligand figures generated by gaff. Ligands are 11r, 13a, 13b, darunavir, lopinavir, and indinavir. Figure S4: The partial charge represented ligand figures generated by charmm-gui. Ligands are x0072, x0689, x0691, x0749, and x1336. Figure S5: The partial charge represented ligand figures generated by charmm-gui. Ligands are 11r, 13a, 13b, darunavir, lopinavir, and indinavir. Figure S6: MM-PBSA decomposed energy per-reisdue with ff99SB (a) X0072 (b) X0689 (c) X0691 (d) X0749 (e) X1336. Figure S7: MM-PBSA decomposed energy per-residue with ff99SB (a) 11r (b) 13a (c) 13b (d) darunavir (e) lopinavir (f) indinavir. Figure S8: MM-PBSA decomposed energy per-residue with c36 (a) X0072 (b) X0689 (c) X0691 (d) X0749 (e) X1336. Figure S9: MM-PBSA decomposed energy per-residue with c36 (a) 11r (b) 13a (c) 13b (d) darunavir (e) lopinavir (f) indinavir. Figure S10: MM-PBSA Decomposed energy per-residue with g54a7 (a) X0072 (b) X06889 (c) X0691 (d) X0749 (e) X1336. Figure S11: MM-PBSA Decomposed energy per-residue with g54a7 (a) 11r (b) 13a (c) 13b (d) darunavir (e) lopinavir (f) indinavir. Figure S12: Hydrogen bond existence map with ff99SB. The scatter plots show a formed hydrogen bond at corresponding frame. Figure S13: Hydrogen bond existence map with c36. The scatter plots show a formed hydrogen bond at corresponding frame. Figure S14: Hydrogen bond existene map with g54a7. The scatter plots show a formed hydrogen bond at corresponding frame. Figure S15: 3D histogram from the production trajectories with ff99sb. Figure S16: 3D histogram from the production trajectories with c36. Figure S17: 3D histogram from the production trajectories with g54a7. Figure S18: The protein-ligand interaction plot generated by ligPlot+ program with ff99sb force field. Figure S19: The protein-ligand interaction plot generated by ligPlot+ program with c36 force field. Table S1: Each energetic components of ΔG component (kcal/mol) of each ligand-M pro calculated by the MM-PBSA analysis.

## Abbreviations

The following abbreviations are used in this manuscript:

FDA	Food and Drug Administration
3CLP	3C-like proteinase
MD simulation	Molecular Dynamics simulation
MM-PBSA	Molecular Mechanics Poisson–Boltzmann Surface Area
ff14sb	Amber14sb force field
c36	Charmm36 force field
g54a7	Gromos54a7 force field
