# Supplementary material for: Identifying the Hot Spot Residues of the SARS-CoV-2 Main Protease Using MM-PBSA and Multiple Force Fields
Source: Life (Basel). 2021 Dec 31;12(1):54. doi: 10.3390/life12010054 (PMC8779590; doi:10.3390/life12010054)
Supplement: Supplementary file 1 [file life-12-00054-s001.zip › life-1502327-supplementary.pdf]

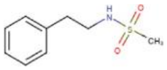

X0072

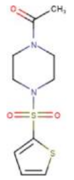

X0689

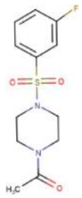

X0691

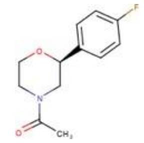

X0749

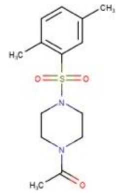

X1336

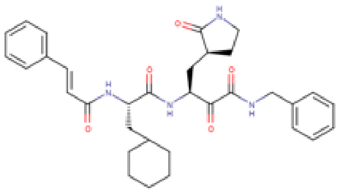

11r

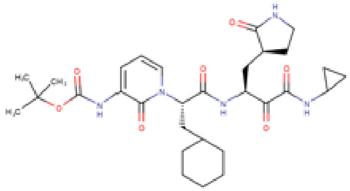

13a

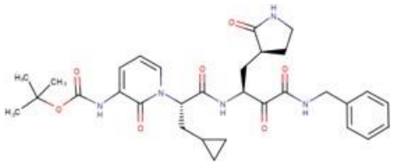

13b

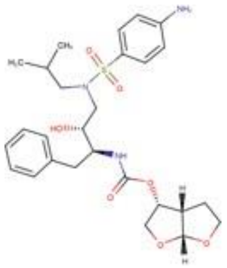

Darunavir

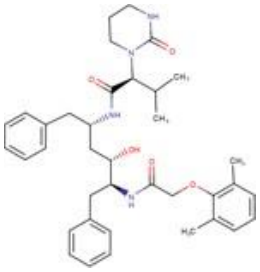

Lopinavir

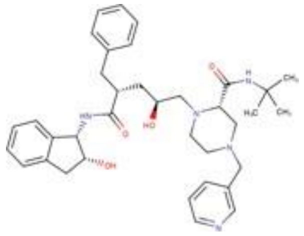

Indinavir

Figure S1. The used ligands in this work.

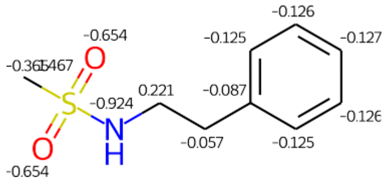

x0072

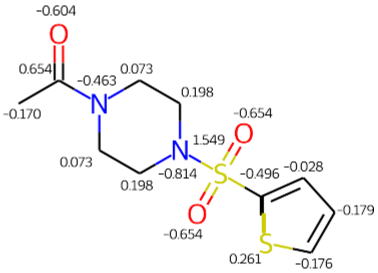

x0689

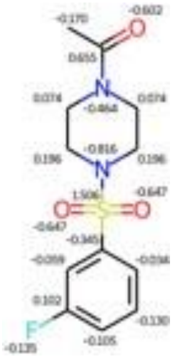

x0691

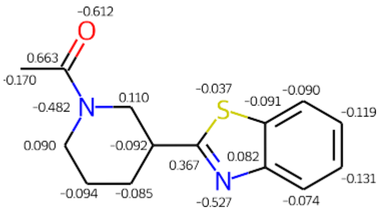

x0749

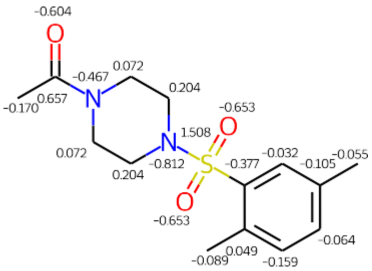

x1336

Figure S2. The partial charge represented ligand figures generated by gaff. Ligands are x0072, x0689,x0691, x0749, and x1336

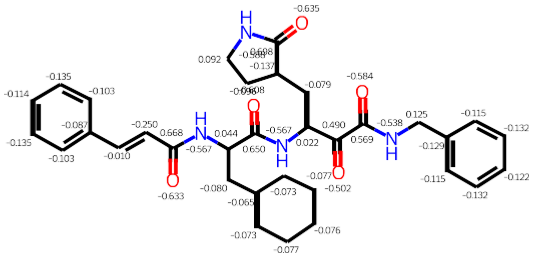

11r

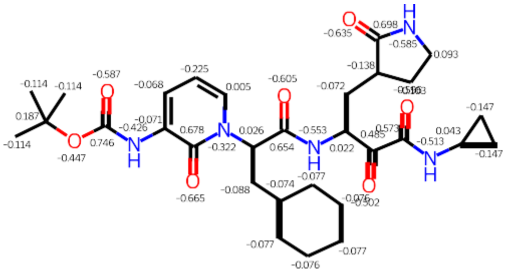

13a

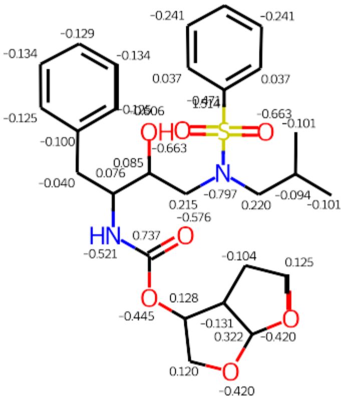

darunavir

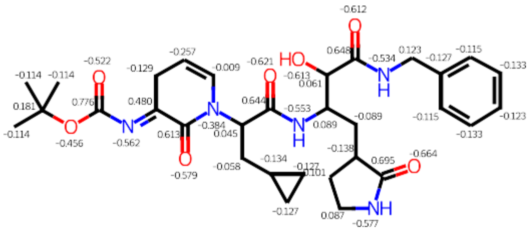

13b

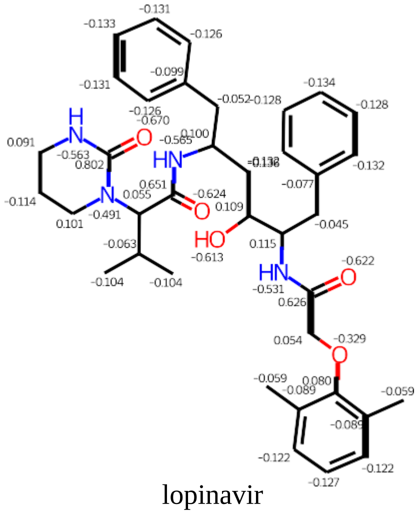

lopinavir

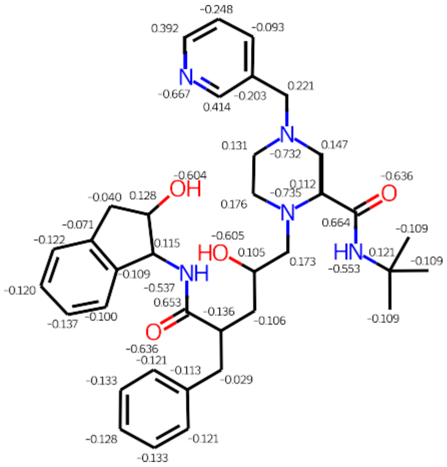

indinavir

Figure S3. The partial charge represented ligand figures generated by gaff. Ligands are 11r, 13a, 13b,darunavir, lopinavir, and indinavir

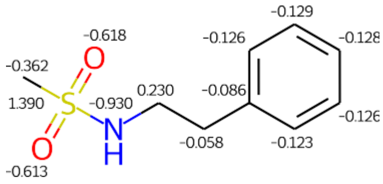

x0072

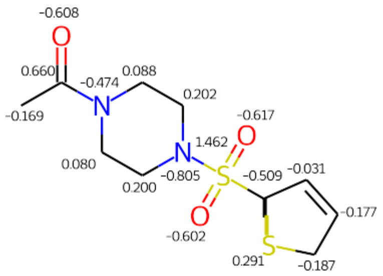

x0689

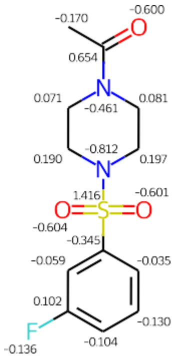

x0691

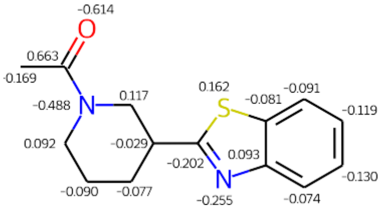

x0749

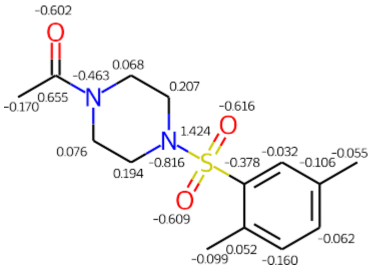

x1336

Figure S4. The partial charge represented ligand figures generated by charmm-gui. Ligands are x0072,x0689, x0691, x0749, and x1336

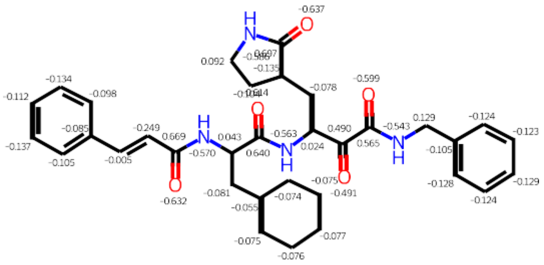

11r

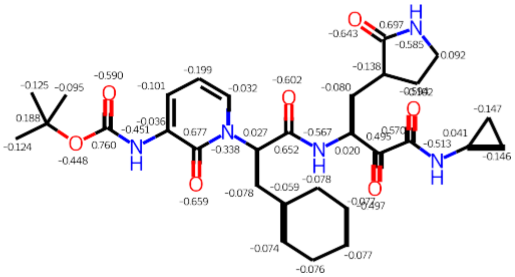

13a

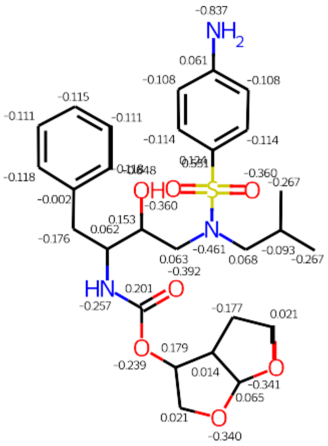

darunavir

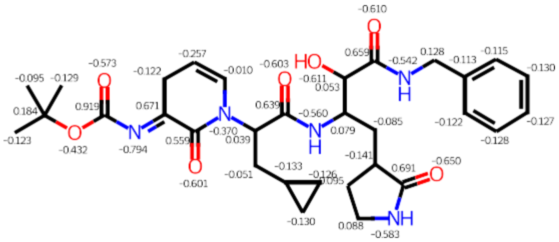

13b

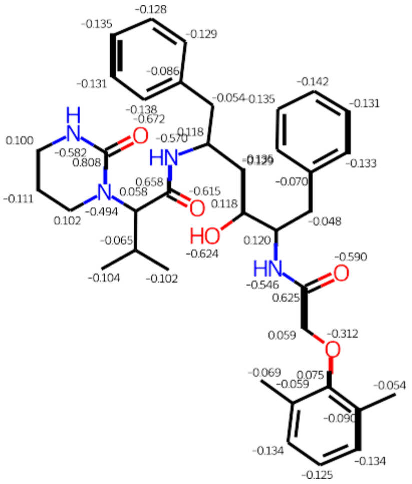

lopinavir

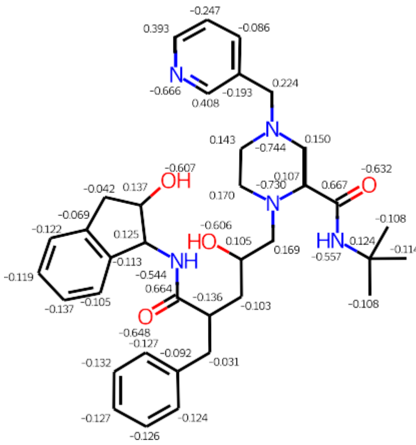

indinavir

Figure S5. The partial charge represented ligand figures generated by charmm-gui. Ligands are 11r, 13a,13b, darunavir, lopinavir, and indinavir

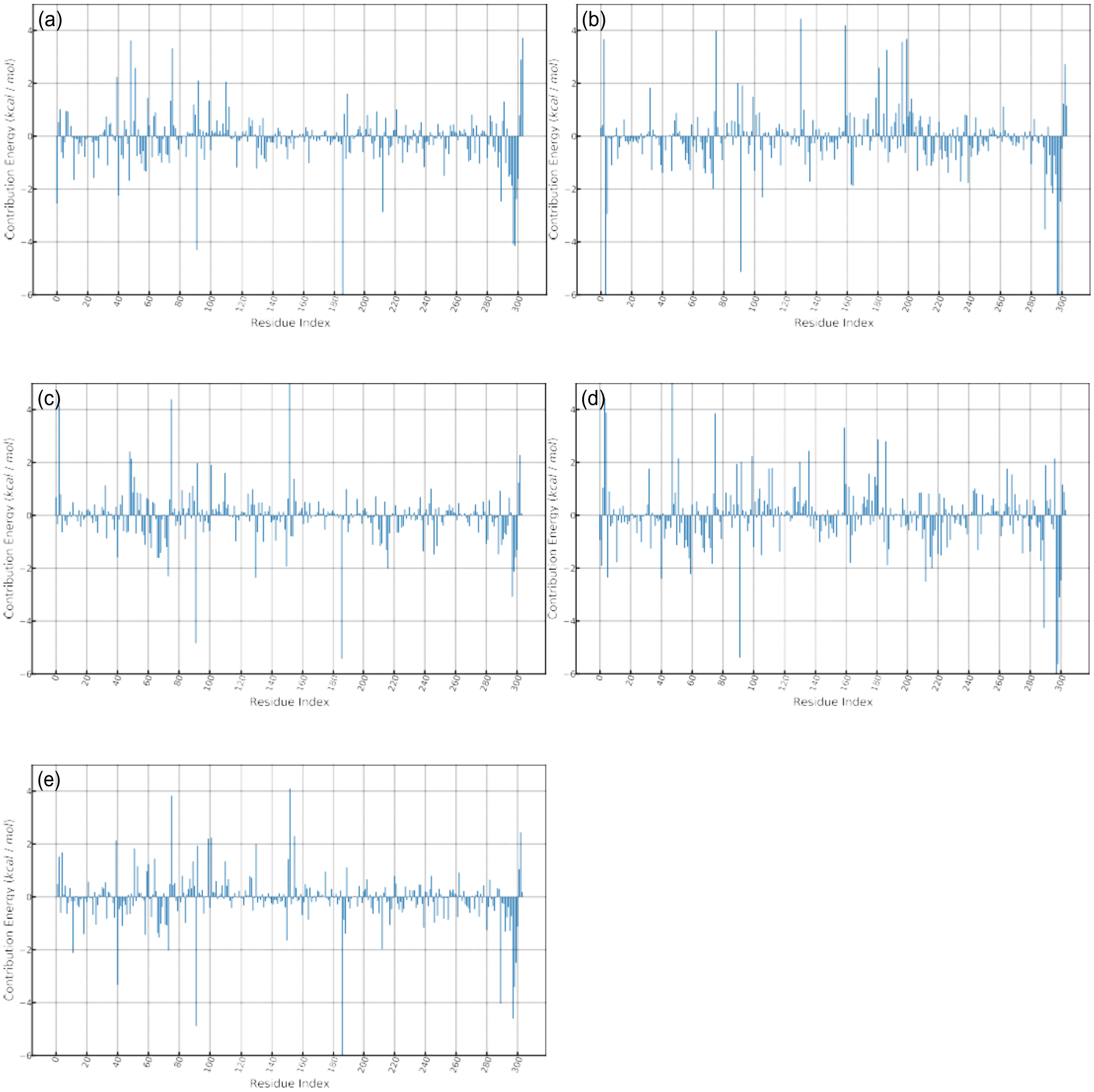

Figure S6. MM-PBSA decomposed energy per-reisdue with ff99SB (a) X0072 (b) X0689 (c) X0691 (d)X0749 (e) X1336

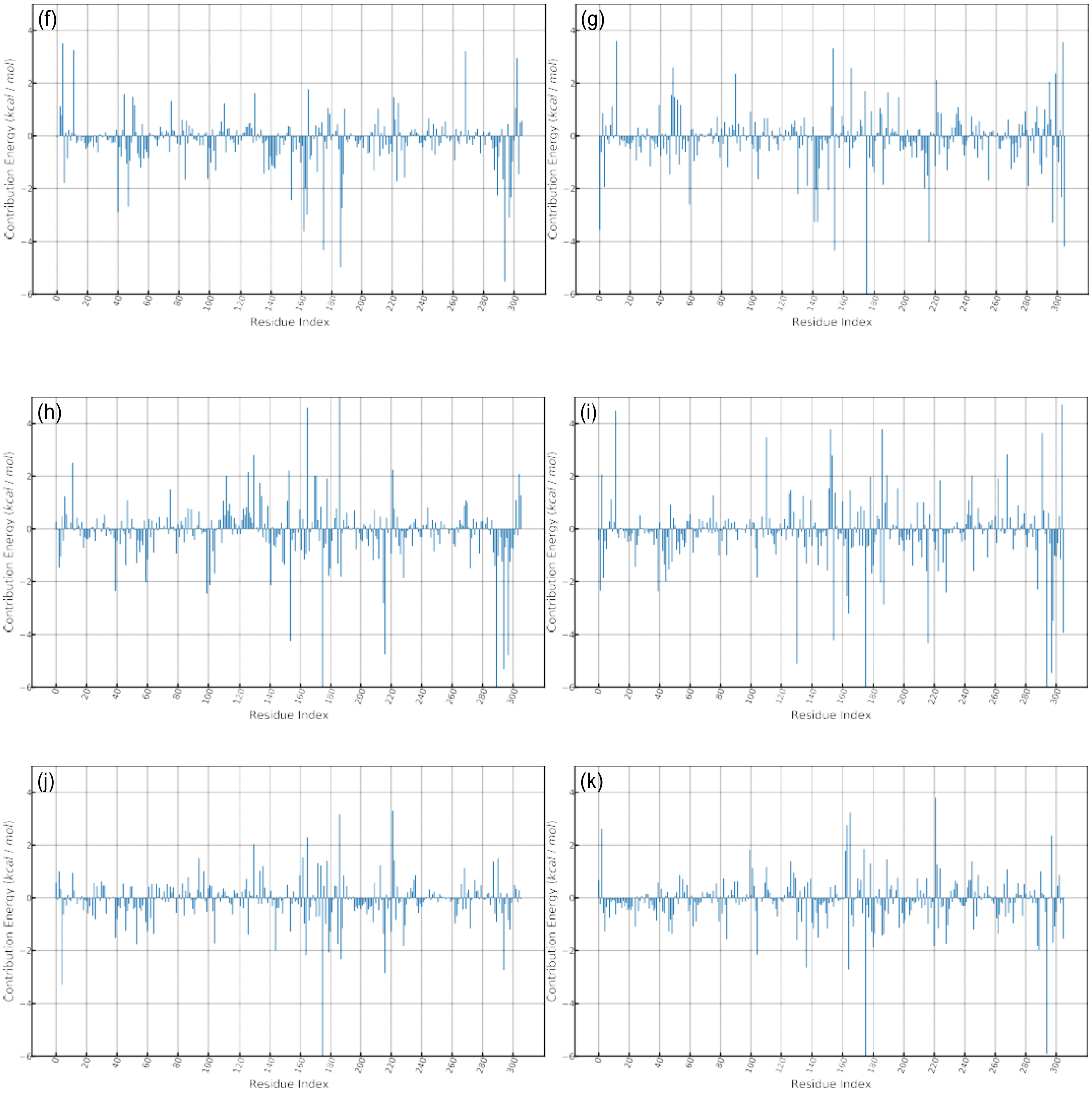

Figure S7. MM-PBSA decomposed energy per-residue with ff99SB (f) 11r (g) 13a (h) 13b (i) darunavir (j) lopinavir (k) indinavir

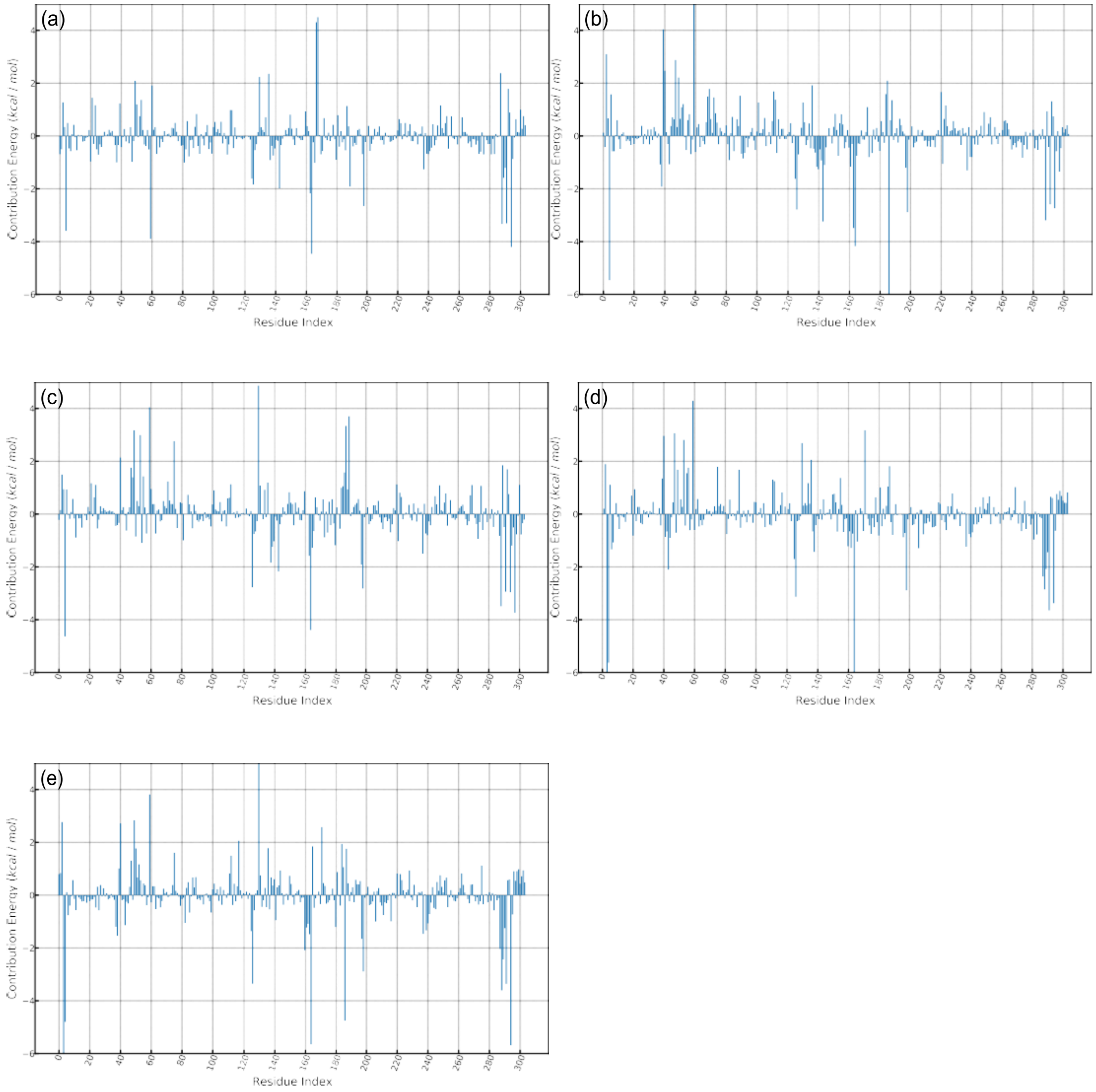

Figure S8. MM-PBSA decomposed energy per-reisdue with c36 (a) X0072 (b) X0689 (c) X0691 (d)X0749 (e) X1336

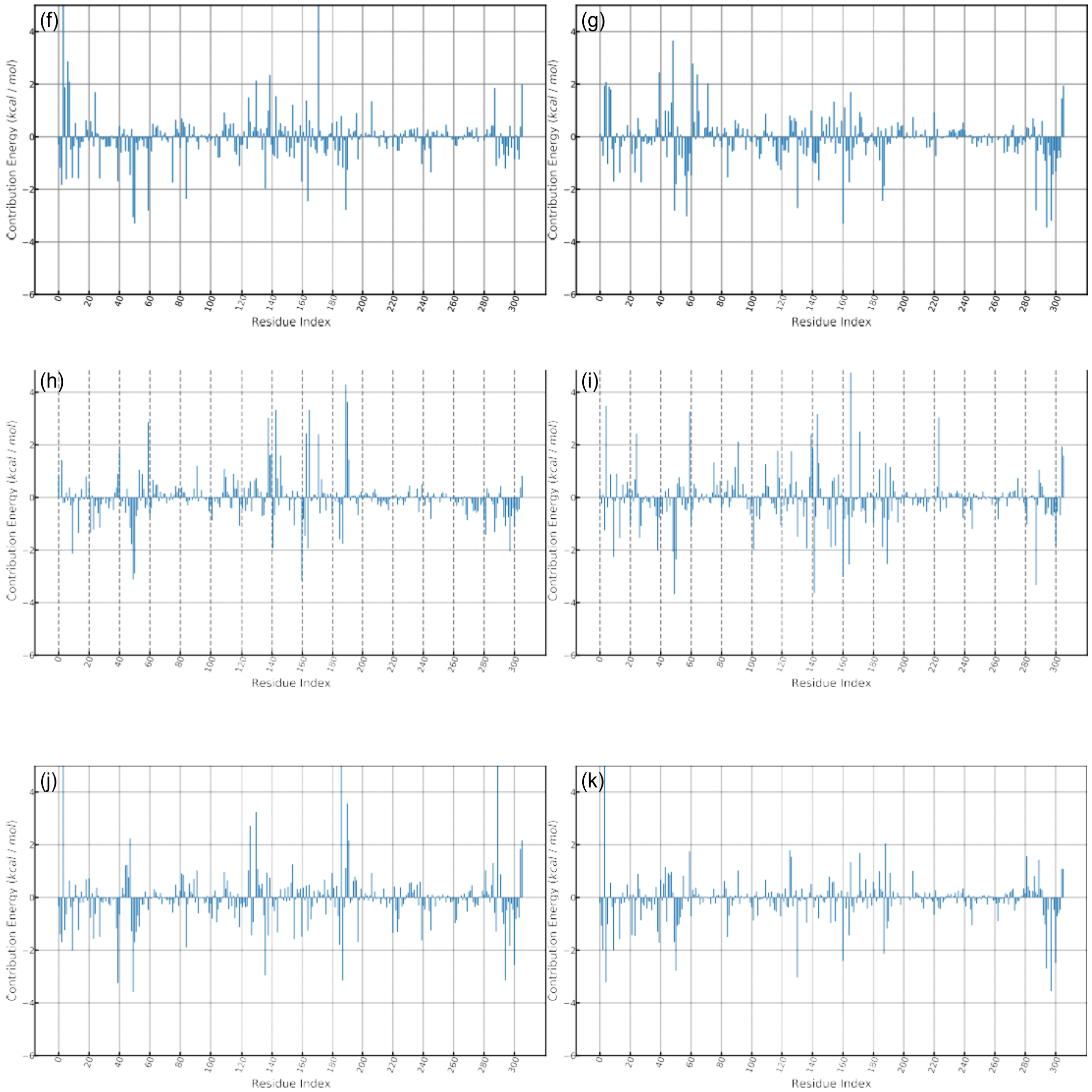

Figure S9. MM-PBSA decomposed energy per-residue with c36 (f) 11r (g) 13a (h) 13b (i) darunavir (j)lopinavir (k) indinavir

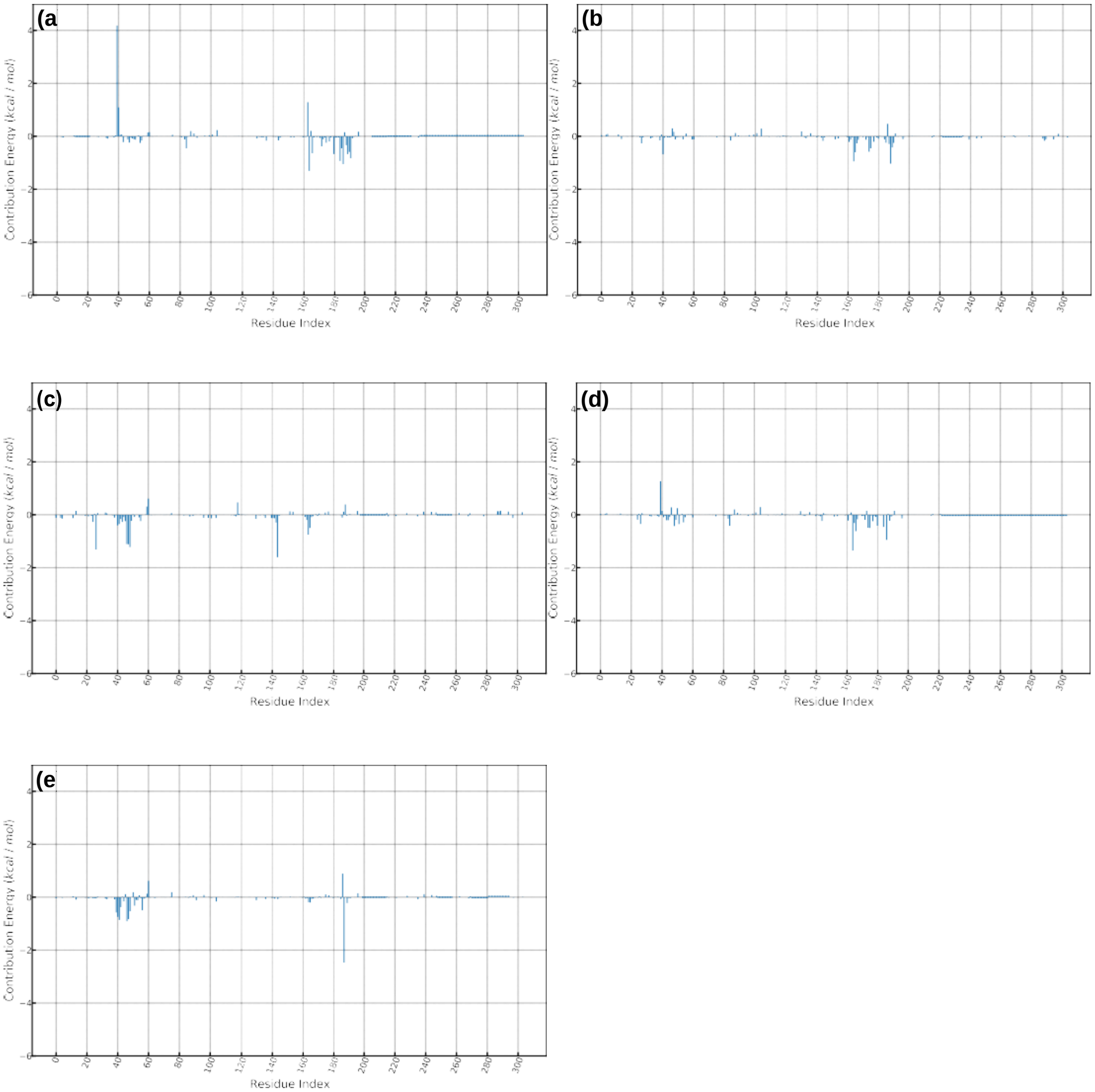

Figure S10. MM-PBSA Decomposed energy per-residue with g54a7 (a) X0072 (b) X06889 (c) X0691(d) X0749 (e) X1336

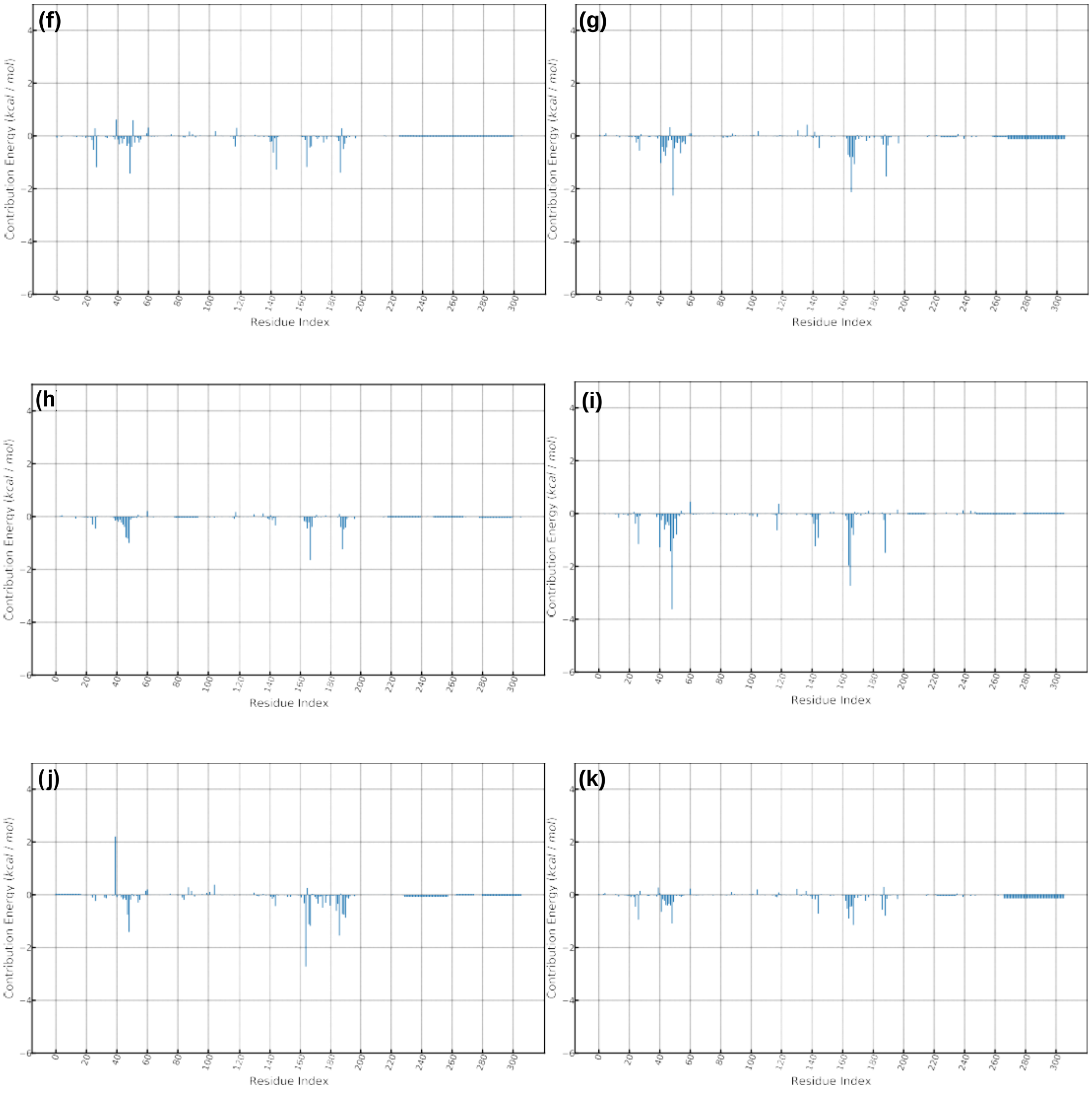

Figure S11. MM-PBSA Decomposed energy per-residue with g54a7 (f) 11r (g) 13a (h) 13b (i) darunavir (j) lopinavir (k) indinavir

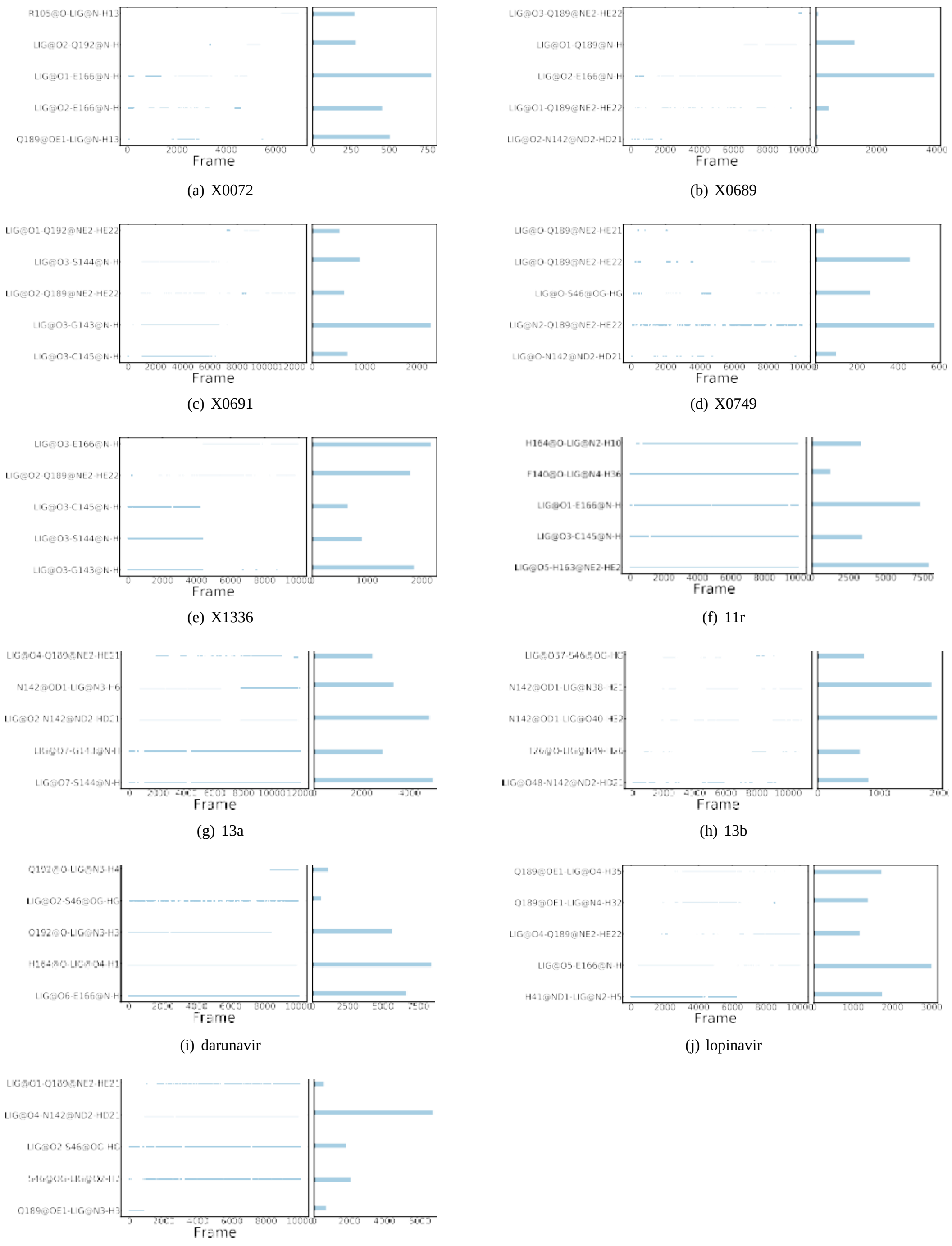

(a) X0072
(b) X0689
(c) X0691
(d) X0749
(e) X1336
(f) 11r
(g) 13a
(h) 13b
(i) darunavir
(j) lopinavir
(k) indinavir

Figure S12. Hydrogen bond existence map with ff99SB

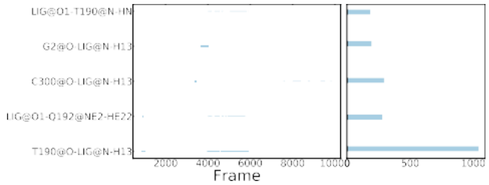

(a) X0072

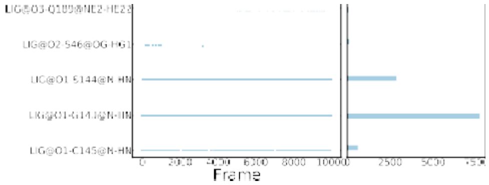

(b) X0689

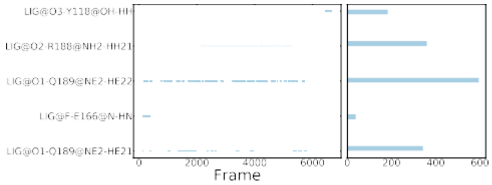

(c) X0691

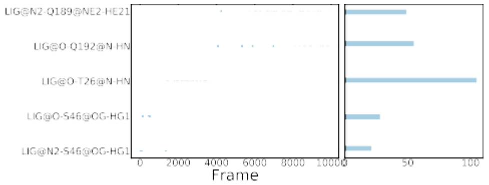

(d) X0749

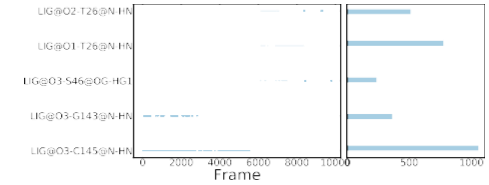

(e) X1336

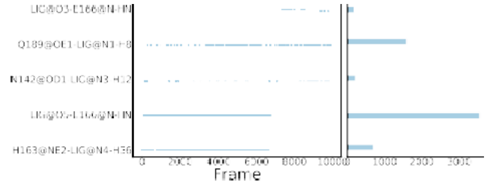

(f) 11r

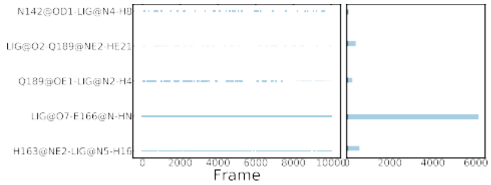

(g) 13a

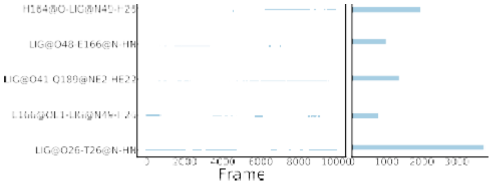

(h) 13b

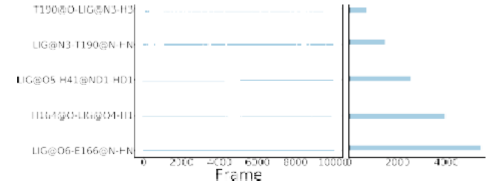

(i) darunavir

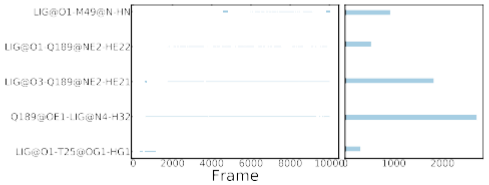

(j) lopinavir

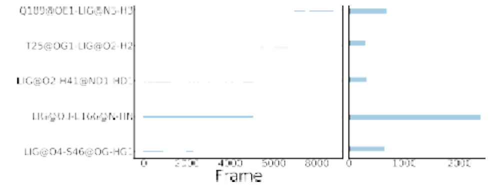

(k) indinavir

Figure S13. Hydrogen bond existence map with c36

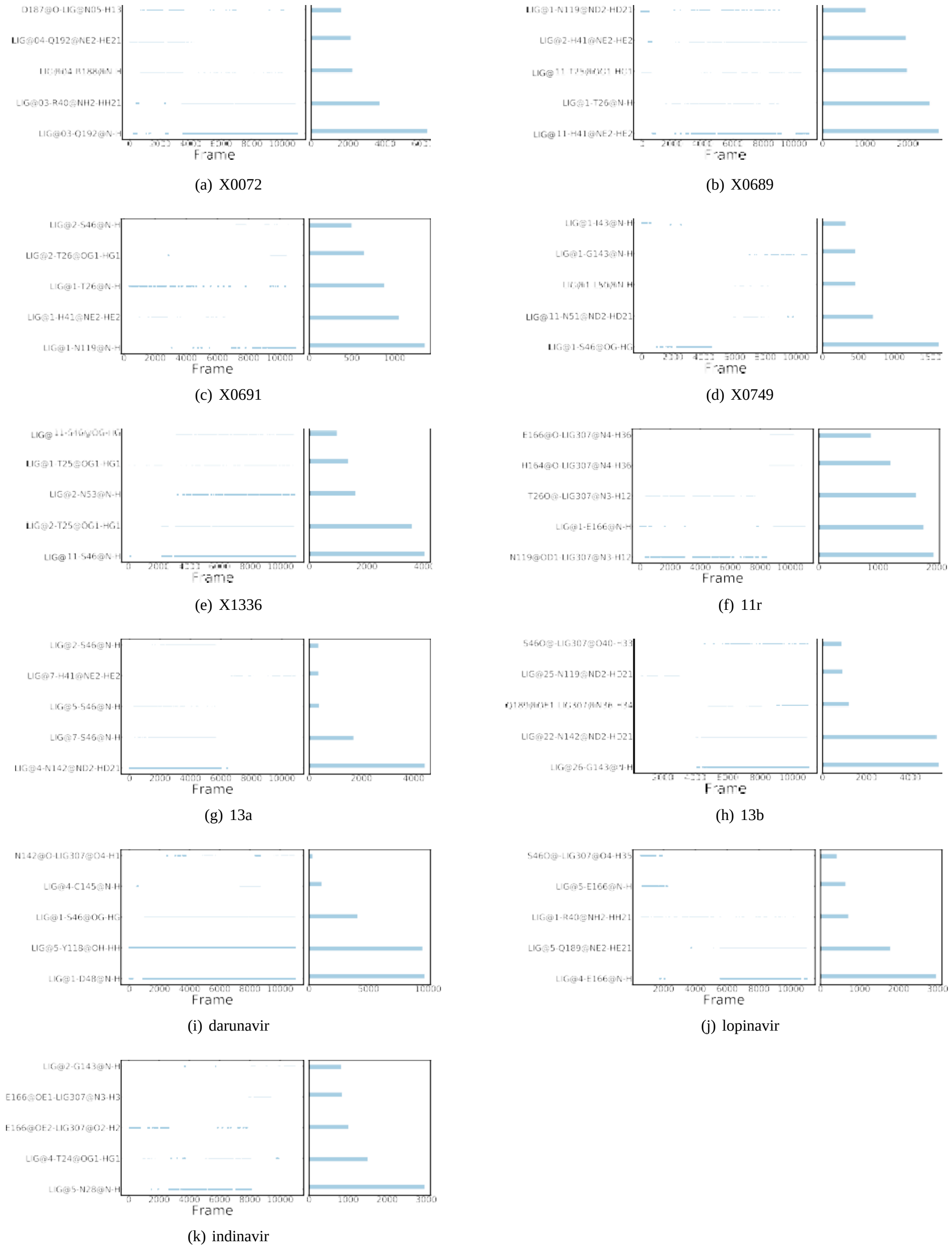

(a) X0072 (b) X0689

(c) X0691 (d) X0749

(e) X1336 (f) 11r

(g) 13a (h) 13b

(i) darunavir (j) lopinavir

(k) indinavir

Figure S14. Hydrogen bond existene map with g54a7

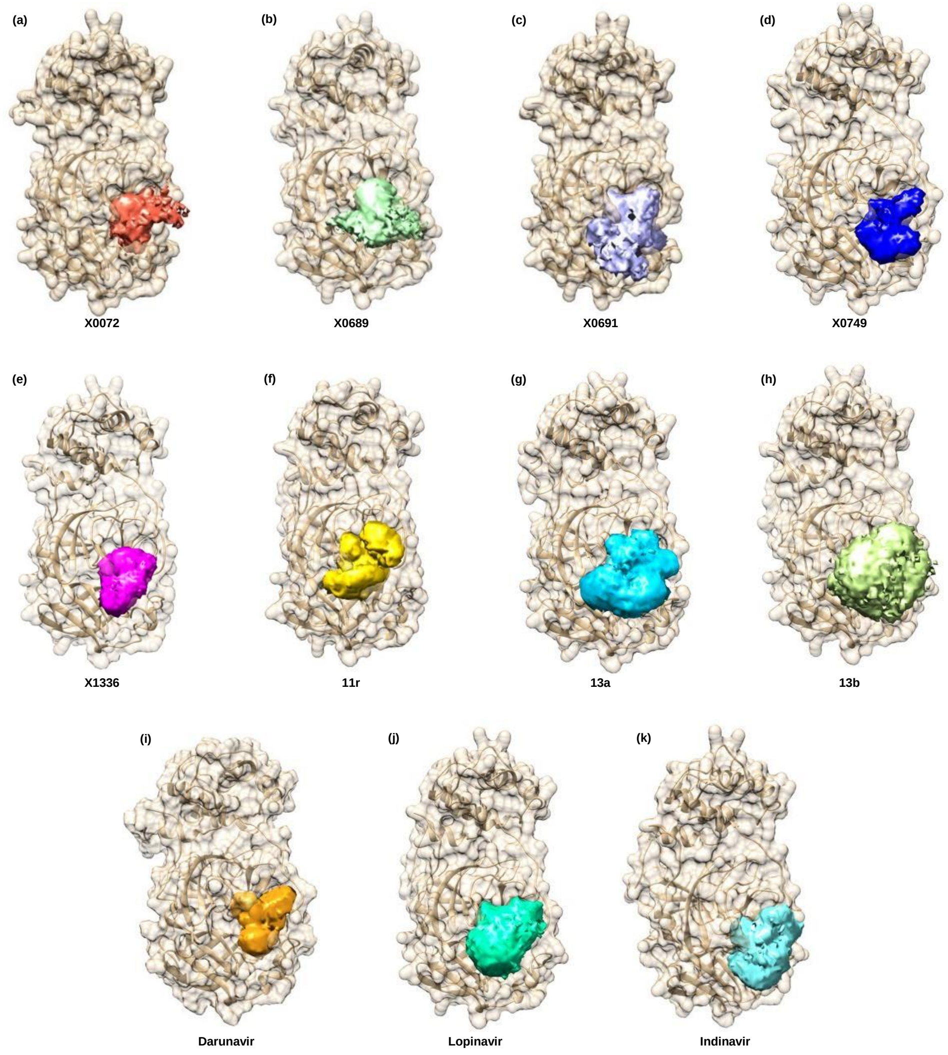

Figure S15. 3D histogram from the production trajectories with ff99SB

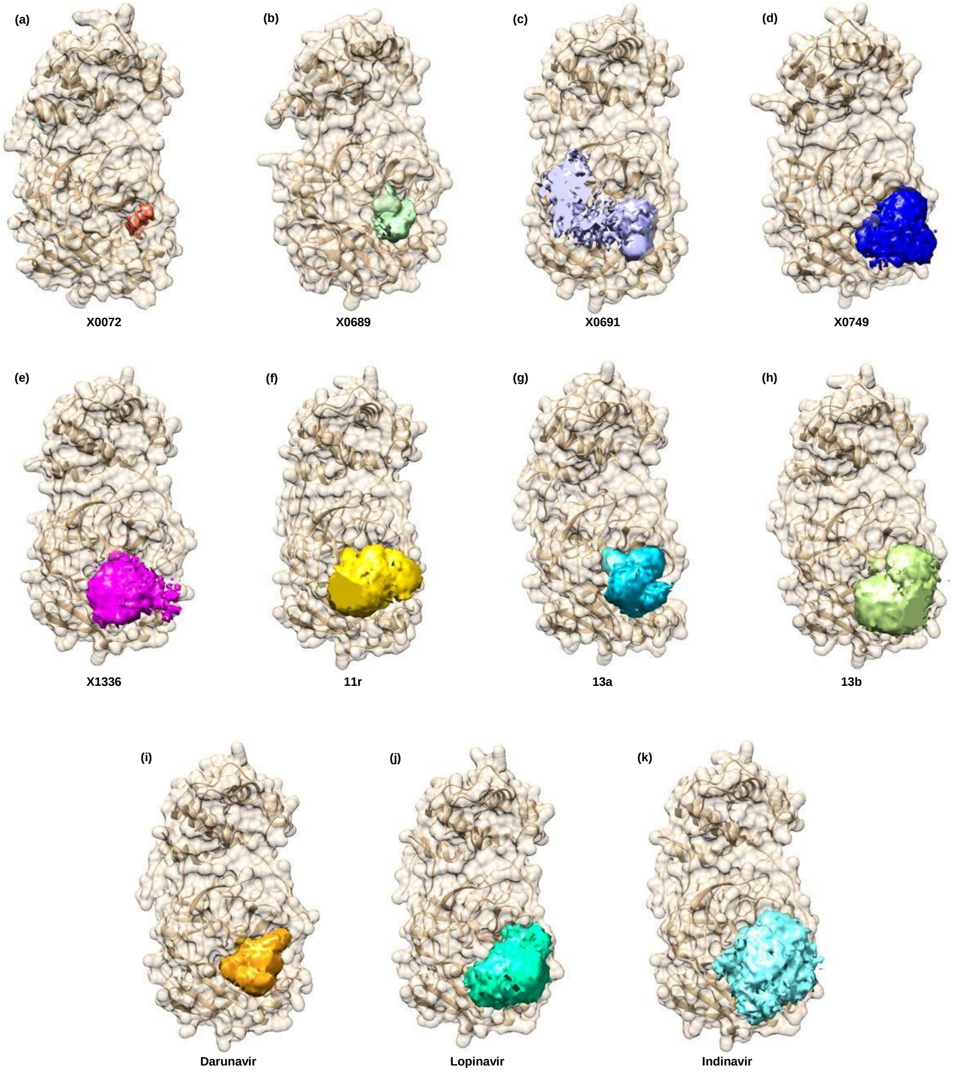

Figure S16. 3D histogram from the production trajectories with c36

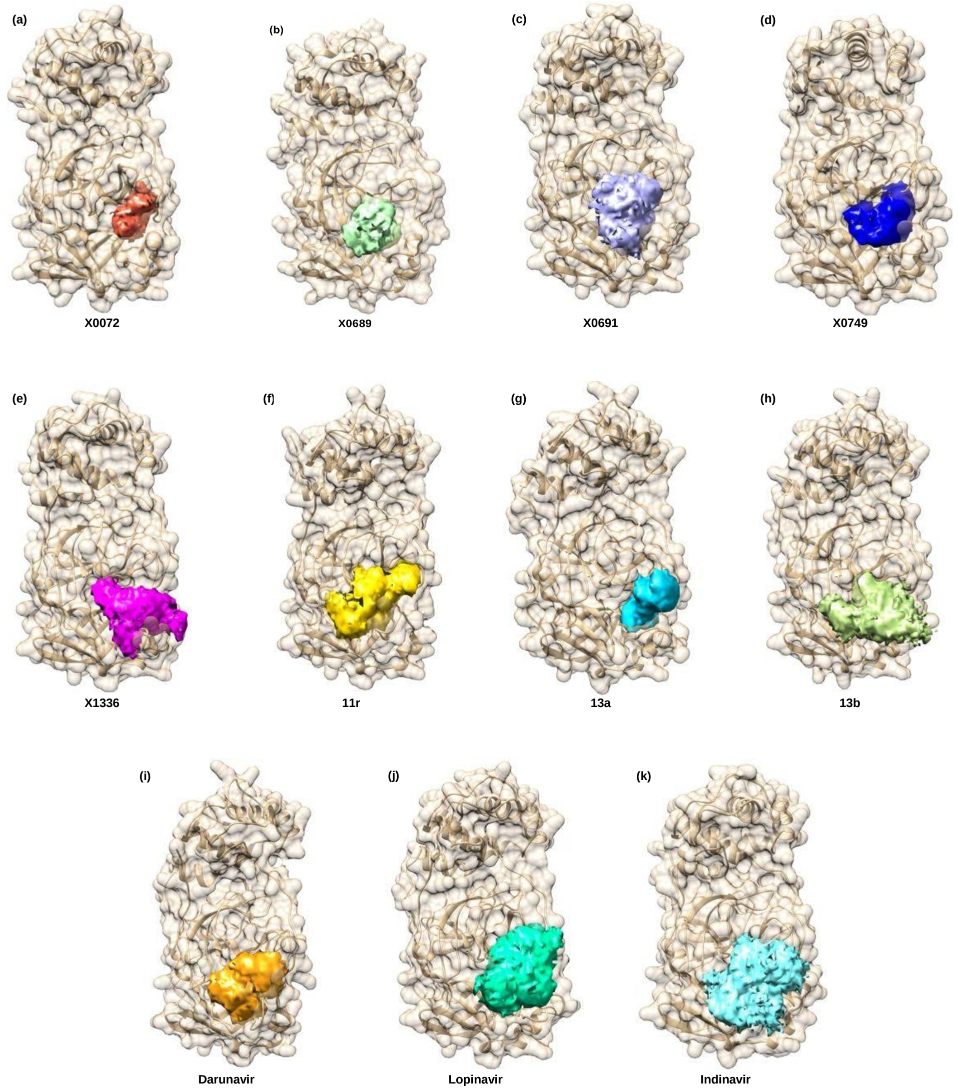

Figure S17. 3D histogram from the production trajectories with g54a7

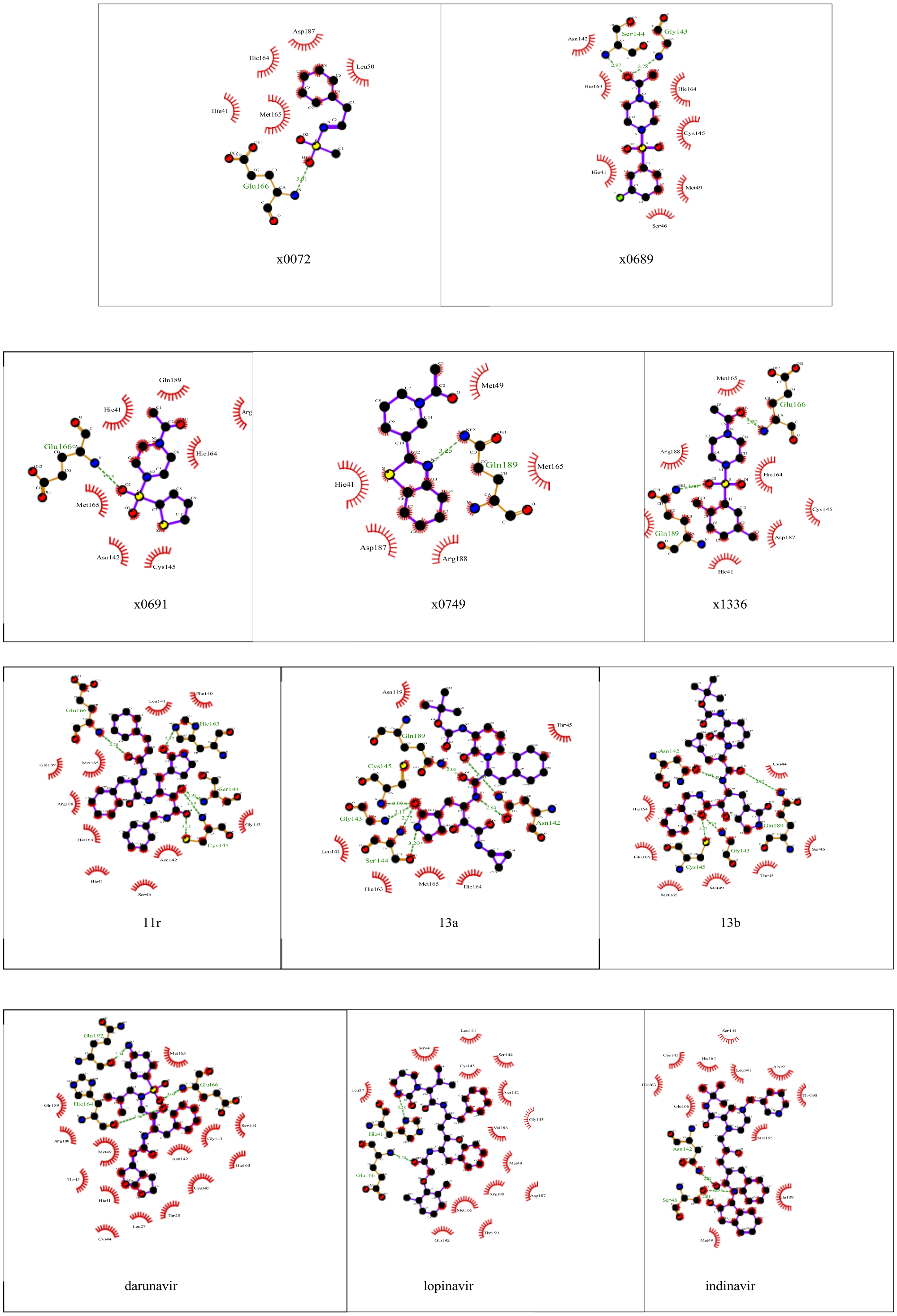

Figure S18. The protein-ligand interaction plot generated by ligPlot+ program with ff99sb force field

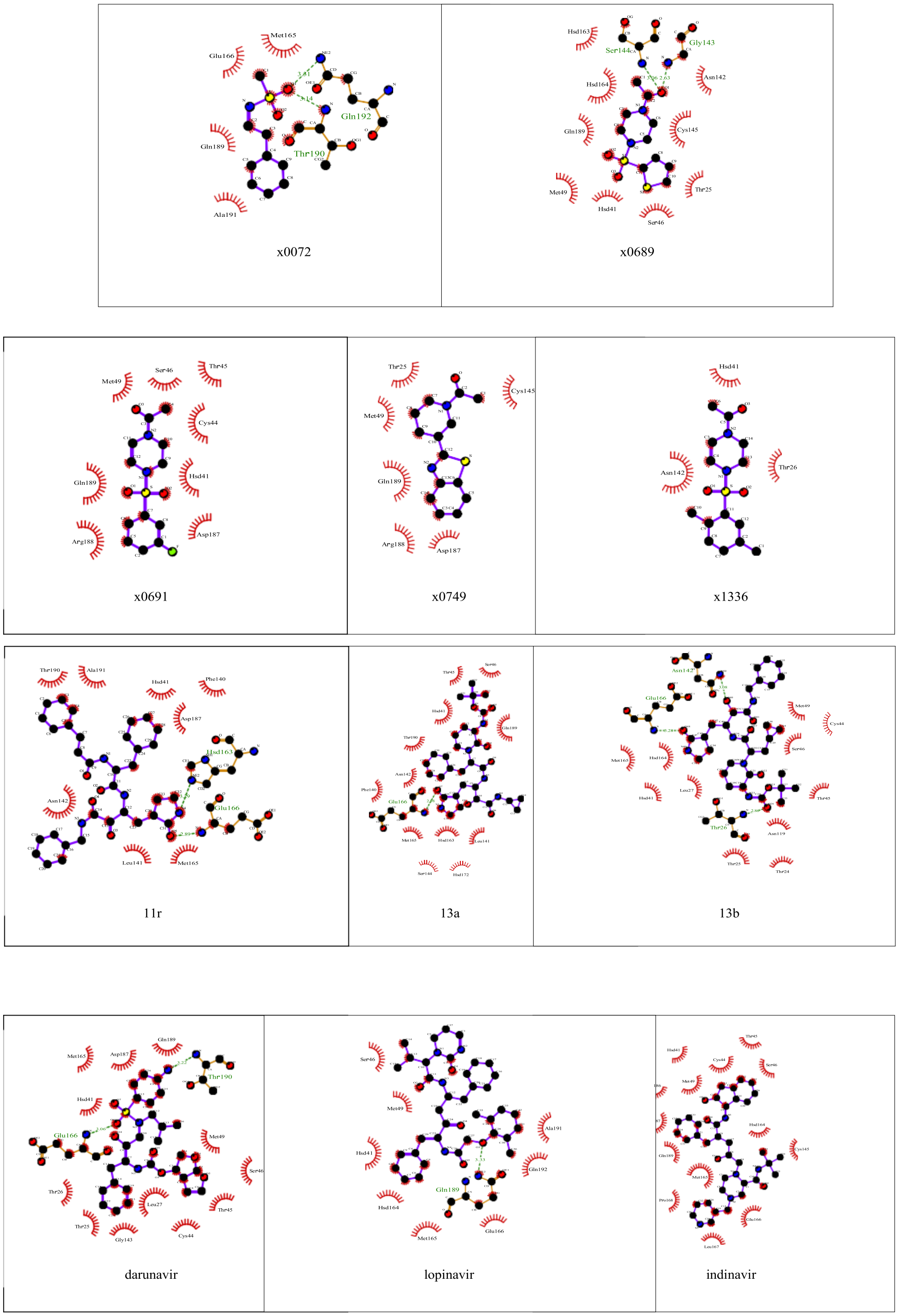

Figure S19. The protein-ligand interaction plot generated by ligPlot+ program with c36 force field

Table S1. Each energetic components of Δ$G_{component}$ (kcal/mol) of each ligand-M^{pro} calculated by the MM-PBSA analysis.

Ligand	E_{vdw}	E_{elec}	E_{MM}	E_{PB}	E_{nonpol}	E_{sol}	E_{total}
**X0072**							
ff99SB	-34.56	-21.98	-55.91	-14.49	35.54	21.05	-34.86
c36	-12.54	-75.34	-83.03	35.95	30.18	66.13	-17.30
g54a7	-31.61	-17.17	-48.78	38.24	-3.12	35.12	-13.66
**X0689**							
ff99SB	-50.56	-111.34	-154.66	69.21	62.23	131.44	-23.22
c36	-20.07	38.12	12.66	-41.79	33.60	-8.19	3.94
g54a7	-30.54	-17.38	-47.92	28.61	-2.92	25.68	-22.24
**X0691**							
ff99SB	-26.76	-13.66	-47.07	3.85	34.14	38.00	-9.08
c36	-11.19	-2.63	-17.93	7.49	23.60	31.09	12.18
g54a7	-28.19	-14.32	-42.51	24.83	-2.87	21.96	-20.55
**X0749**							
ff99SB	-42.95	-20.21	-62.54	-8.86	53.23	44.37	-18.17
c36	-31.06	-53.27	-92.13	34.89	44.00	78.89	-13.24
g54a7	-38.32	-6.22	-44.54	26.06	-3.56	22.50	-22.04
**X1336**							
ff99SB	-37.13	24.50	-18.83	-51.88	46.99	-4.89	-23.73
c36	-7.08	-47.02	-63.11	28.08	26.98	55.07	-8.93
g54a7	-31.97	-23.57	-55.55	35.98	-3.17	32.81	-22.73
**11r**							
ff99SB	-68.82	-24.22	-91.33	-37.34	73.68	36.34	-54.99
c36	-32.06	-14.22	-46.84	-12.54	49.77	37.23	-10.18
g54a7	-53.31	-11.87	-65.18	33.43	-6.16	27.27	-37.91
**13a**							
ff99SB	-43.84	-97.48	-146.15	51.74	56.44	108.18	-37.97
c36	-27.47	-53.46	-69.46	-3.84	44.60	40.76	-28.52
g54a7	-56.01	-14.01	-70.02	31.60	-5.19	26.41	-43.61
**13b**							
ff99SB	-35.98	-17.60	-52.33	-25.44	48.24	22.80	-29.54
c36	-35.20	-138.26	-171.51	116.50	60.66	177.16	5.58
g54a7	-38.31	-18.49	-56.80	29.35	-4.48	24.87	-31.93
**DARUNAVIR**							
ff99SB	-57.96	-50.70	-110.38	-0.73	60.82	60.10	-50.29
c36	-44.52	-90.10	-124.84	58.39	64.57	122.96	-1.56
g54a7	-52.76	-23.14	-75.90	34.72	-5.45	29.26	-46.64
**INDINAVIR**							
ff99SB	-67.49	11.94	-54.86	-49.95	75.58	25.62	-29.24
c36	-33.10	-20.36	-59.65	-3.66	42.03	38.37	-21.34
g54a7	-44.27	-13.41	-57.68	26.13	-4.59	21.54	-36.14
**LOPINAVIR**							
ff99SB	-53.21	10.14	-35.53	-47.76	66.23	18.47	-17.06
c36	-36.66	-33.91	-63.79	-1.39	52.52	51.13	-13.17
g54a7	-53.32	-11.17	-64.49	29.31	-5.57	23.74	-40.74
